# Mitochondria-localized photocatalyst of biomimetic organic semiconductor nanoparticles for NIR-activatable photocatalytic immunotherapy

**DOI:** 10.1126/sciadv.adx4850

**Published:** 2025-12-05

**Authors:** Yu Wang, Yijian Gao, Yuliang Yang, Jie Zhang, Yingpeng Wan, Xiliang Li, Ning Li, Guangtao Fu, Yuhui Yang, Zeng Li, Qi Zhao, Yuanfeng Chen, Shengliang Li

**Affiliations:** ^1^ College of Pharmaceutical Sciences, The Fourth Affiliated Hospital of Soochow University, Suzhou Medical College, Soochow University, Suzhou 215123, P. R. China.; ^2^ Department of Orthopedics, Guangdong Provincial People’s Hospital (Guangdong Academy of Medical Sciences), Southern Medical University, Guangzhou, 510100, P. R. China.; ^3^ Research Department of Medical Science, Guangdong Provincial People’s Hospital (Guangdong Academy of Medical Sciences), Southern Medical University, Guangzhou 510100, P.R. China.

## Abstract

Photocatalytic therapy holds great prospects in high-performance tumor treatment owing to its advantages of noninvasiveness and oxygen nonreliance. However, there is still a lack of ideal photocatalyst materials that respond to near-infrared (NIR) light, especially with organelle-specific features. Here, a mitochondria-biomimetic organic semiconductor photocatalyst was designed for NIR-activatable photocatalytic immunotherapy of recurrent and metastatic tumor. With biomimetic of mitochondrial membrane, a NIR-responsive conjugated polymer YBSe-SS was prepared into biomimetic nano-photocatalysts (Mito-NPs). Upon NIR-light irradiation, the Mito-NPs achieved effective photocatalytic oxidation of reduced nicotinamide adenine dinucleotide and mitochondria dysfunction, benefiting from the mitochondria-targeted performance. Also, relatively high multiphototheranostics were demonstrated by the Mito-NPs. Subsequently, with Mito-NP–regulated mitochondrial function and metabolism, the in vivo experiments proved that the photocatalytic Mito-NPs combined with immune checkpoint therapy achieved photocatalytic immunotherapy for suppressing primary and pulmonary metastasis of osteosarcoma. This work offers a practical paradigm for NIR-activatable organic photocatalytic materials for high-performance tumor therapy.

## Teaser

NIR-responsive biomimetic nano-photocatalysts (Mito-NPs) with mitochondria targeting achieve photocatalytic tumor immunotherapy.

## INTRODUCTION

Phototherapy mainly including photothermal therapy (PTT) and photodynamic therapy (PDT) has become a highly-anticipated paradigm of precision cancer therapy, benefiting from its advantages of noninvasive, nontolerability, small side effects, and convenient operation (*1*–*4*). PDT based on local activation of photosensitizers (PSs) generating reactive oxygen species (ROS) can rapidly interact with biomass, resulting in enzyme inactivation, biofilm structure damage, cell death, blood vessel damage, etc. (*5*–*8*), and lastly achieve antitumor effects. In particular, near-infrared (NIR) region (700 to 1700 nm) responsive PSs have deeper tissue penetration, which has been used clinically to treat a variety of cancers, including superficial skin cancer and esophageal cancer (*9*–*14*). Meanwhile, PDT is also easily combined with other treatments to form multimodal combination therapies, such as PTT, immunotherapy, etc., which can be used to improve the efficiency of tumor tissue ablation and inhibit metastasis and recurrence (*15*–*17*). Recently, organic PSs have been ideal candidates due to their high biocompatibility, easy modification, and excellent photostability, especially excellent optical properties in NIR (*18*–*24*). However, the hypoxic tumor microenvironment seriously limits the validity of conventional organic PSs (*25*–*28*). Thus, it is essential to explore NIR-activated organic PSs with new models of oxygen (O_2_) nonreliance, enhancing the efficiency of eliminating solid tumors (*29*–*31*).

Photocatalytic therapy (PCT) is an emerging cancer therapy strategy that uses photocatalytic reaction to disrupt the redox balance of the tumor cells and then induce cell death (*32*, *33*). PCT is a more advantageous phototherapy mode because of its advantages in noninvasiveness, hypotoxicity, and efficiency in hypoxic tumor, besides the O_2_-independence property (*34*–*36*). Recently, extensive investigations have been made on the exploitation and development of high-performing PCT materials for efficient tumor treatment. For example, Huang and coworkers (*37*–*42*) verified the killing effect of PCT on tumor cells using iridium (Ir) complexes and ruthenium (Ru) complexes as photocatalysts. Peng and Li’s groups (*43*, *44*) found that several well-known organic PSs—such as rose bengal, boron-dipyrromethene, phthalocyanine, and hematoporphyrin—are effective candidates for PCT, achieving desirable intracellular reactions under mild reaction conditions. Recently, Tang and Zhou’s group (*45*) developed an aggregation-induced emission molecule as a photocatalyst for biological applications under visible light excitation. However, these reported photocatalysts are mostly concentrated in short-wavelength excitation (white light or ultraviolet light), which shows severe limitations for tumor therapy in the deep tissue (*46*–*48*). Furthermore, photocatalysts based on metal complexes contain heavy metal elements such as Ir, Ru, zinc, or nickel, exhibiting unpredicted cytotoxicity to living organisms, greatly precluding their widespread use (*49*–*54*). Hence, exploring high-performance long-wavelength–responsive organic photocatalysts for PCT applications is essential. However, developing a material with suitable photocatalytic performance upon NIR excitation is still a great challenge.

Mitochondria are special membrane-bound cell organelles, regarded as the powerhouse of eukaryotic cells (*55*–*57*). Recent studies indicate that mitochondrial dysfunction is implicated in a wide range of diseases, including cancers. In addition to providing the energy necessary to sustain metabolism, mitochondria participate in a multitude of cellular processes including signaling transmission, calcium homeostasis, production of ROS, and cellular apoptosis cascade reaction, which then leads to a series of biological effects, such as apoptosis, pyroptosis, autophagy, immunogenic death, etc. (*58*, *59*). Because mitochondria play crucial roles in regulating cell survival, they have been regarded as popular targets in mitochondria-based disease therapy (*60*–*62*). Numerous studies about mitochondria-manipulating techniques of nanomedicines have been developed to regulate mitochondrial function and relative biological effects (*63*–*69*). Therefore, an efficient mitochondria-manipulating technique is a potentially important strategy for high-quality tumor therapy.

**Fig. 1.**
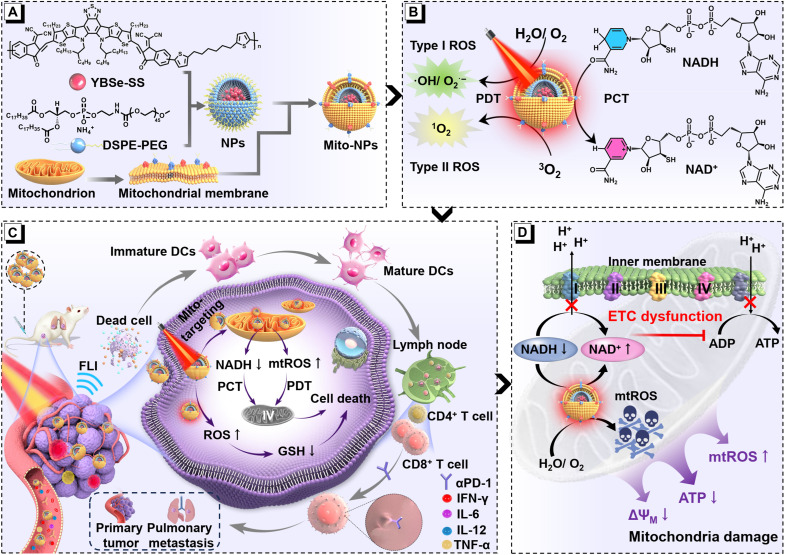
Schematic diagram of the antitumor therapy mechanism. (**A**) Preparation process of Mito-NPs. (**B**) Photophysical Properties of Mito-NPs triggered by NIR photoactivation. (**C**) NIR-II fluorescence imaging-guided photoimmunotherapy anticancer mechanism for primary and lung metastatic osteosarcoma under NIR light irradiation. (**D**) Detailed schematic of the mechanism of photoinduced mitochondrial damage in Mito-NPs. Created in BioRender. Wang, Y. (2025) https://BioRender.com/9pl54jw.

To overcome the above limitations, a biomimetic organic semiconductor photocatalyst was designed for mitochondria-targeted NIR-activatable photocatalytic immunotherapy of protopathic, recurrent, and metastatic tumor (Fig. 1). With the mitochondria-biomimetic technique, a NIR-responsive conjugated polymer YBSe-SS was prepared into biomimetic nano-photocatalysts (Mito-NPs) to obtain the photocatalyst. Upon irradiation by 808-nm light, the Mito-NPs exhibited a relatively high photothermal conversion efficiency (PCE, η) of 80.3 and 2.7% of NIR-II fluorescence quantum yield (QY) with type I and type II ROS generation. Notably, the Mito-NPs were found to have mitochondria-targeted performance and thus achieved effective photocatalytic oxidation of reduced nicotinamide adenine dinucleotide (NADH) and mitochondria dysfunction. Function genomics further found that the mitochondria-targeted photocatalytic Mito-NPs could efficiently regulate the mitochondria function and metabolism under 808-nm light excitation. Subsequently, the primary and pulmonary metastasis of the osteosarcoma model was constructed, and the effectiveness of the NIR-II fluorescence imaging guided tumor ablation. The photocatalytic Mito-NPs combined with immune checkpoint therapy could produce a good immunological effect and thus achieve photocatalytic immunotherapy to suppress osteosarcoma metastasis. This study provides a previously unidentified paradigm for NIR-activatable organic photocatalytic materials in high-performance tumor therapy.

## RESULTS

### Mito-NPs preparation and characteristics

**Fig. 2.**
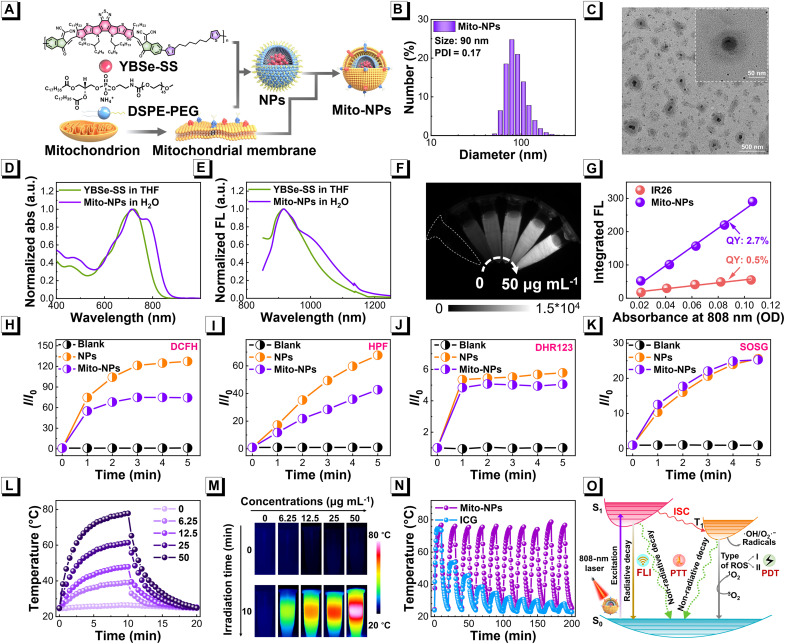
Preparation and characteristics of Mito-NPs. (**A**) Schematic illustration of the preparation of Mito-NPs. (**B**) Size distribution of Mito-NPs. (**C**) TEM image of Mito-NPs. Normalized absorption (**D**) and fluorescence emission (**E**) spectra of Mito-NPs and YBSe-SS. (**F**) NIR-II fluorescence image of Mito-NPs at different concentrations upon 808-nm light excitation. (**G**) Plots of the integrated fluorescence and QY of Mito-NPs in water and IR26 in 1,2-dichloroethane. Plots of relative fluorescence change of DCFH (**H**), HPF (**I**), DHR123 (**J**), and SOSG (**K**) for general ROS, •OH, ${O_{2}}^{•-}$, and O21 detection after treated with Mito-NPs (50 μg ml^−1^) and pristine NPs (50 μg ml^−1^) under 808-nm laser irradiation (0.33 W cm^−2^). (**L**) Photothermal curves of Mito-NPs at different concentrations of 0 to 50 μg ml^−1^ under 1 W cm^−2^ irradiation of 808-nm laser. (**M**) Infrared thermal images of Mito-NPs. (**N**) PTT consistency of Mito-NPs and indocyanine green (ICG) under 10 cycles of laser irradiation (1 W cm^−2^). (**O**) Schematic illustration of multiple NIR-responsive photoactivities of Mito-NPs upon 808-nm excitation.

A NIR-responsive photocatalytic conjugated polymer YBSe-SS was synthesized via Knoevenagel condensation and Stille polymerization (fig. S1). The intermediate and end product were well confirmed by ^1^H nuclear magnetic resonance (NMR), ^13^C NMR, and high-resolution mass spectrometry spectra (figs. S2 to S5). The highest occupied molecular orbital (HOMO) and the lowest unoccupied molecular orbital (LUMO) of YBSe-SS were investigated, and the energy gap (*E*_g_) of YBSe-SS was measured to be ~2.0 eV (fig. S6) (*70*, *71*). Also, the YBSe-SS showed a high molar absorption coefficient of 27.3 L g^−1^ cm^−1^ in tetrahydrofuran (THF). To endow with mitochondrial targeting capacity, water-dispersible NPs of YBSe-SS were prepared with 1,2-distearoyl-*sn*-glycero-3-phosphoethanolamine-*N*-[methoxy(polyethylene glycol)-2000] (DSPE-PEG) via a nanoprecipitation method and then coated with isolated mitochondria membrane to obtain Mito-NPs (Fig. 2A). As demonstrated by dynamic light scattering (DLS), the hydrodynamic diameter of Mito-NPs was around 90 nm, which was slightly higher than that of pristine NPs (Fig. 2B and fig. S7). A negative zeta potential (−19.1 mV) was observed for the Mito-NPs (fig. S8). The transmission electron microscopy (TEM) images, SDS–polyacrylamide gel electrophoresis protein electrophoresis, and element mapping of Mito-NPs displayed an obvious membrane structure around the NPs, indicating the successful biomimetic coating of the mitochondrial membrane (Fig. 2C and figs. S9 and S10). Also, Mito-NPs showed good stability in various solutions within 14 days of storage, similar to pristine NPs (figs. S11 and S12). Next, the ultraviolet-visible-NIR absorption of Mito-NPs was characterized, and the result is listed in Fig. 2D. Mito-NPs and their pristine NPs have a similar maximum absorption peak of 775 nm, indicating that mitochondrial membrane coating hardly affected the absorption profile of NPs (fig. S13). The fluorescence emission spectrum of Mito-NPs was investigated and found that the Mito-NPs exhibited an obvious NIR-II fluorescence upon 808-nm excitation, with a typical concentration-dependent profile (Fig. 2, E and F). Moreover, the fluorescence QY of Mito-NPs was calculated to be 2.7%, referring to 4-[2-[2-chloro-3-[2-(2-phenylthiochromen-4-yl)ethenyl]-1-cyclohex-2-enylidene]ethylidene]-2-phenyl-thiochromene (IR26, QY: 0.5% in 1,2-dichloroethane), suggesting their relatively high NIR-II fluorescence efficiency (Fig. 2G), which is comparable to that of previously reported materials (table S1). These results demonstrated the successful preparation of mitochondria-biomimetic Mito-NPs with typical NIR properties.

### NIR-responsive phototherapeutic activities

To evaluate the therapeutic potential, the PDT and PTT properties of Mito-NPs were further investigated under 808-nm light excitation. The ROS generation capacity of Mito-NPs was investigated by multiple indicators, including 2′,7′-dichlorodihydrofluorescein (DCFH) for the total ROS, hydroxyphenyl fluorescein (HPF) and dihydrorhodamine 123 (DHR123) for type-I ROS, singlet oxygen sensor green (SOSG) for type-II ROS, and ROS green H_2_O_2_ probe for H_2_O_2_, respectively. As illustrated in Fig. 2H and fig. S14, the fluorescence signal intensity of DCF was strengthened instantly in the presence of NPs and Mito-NPs along with the radiation time. Also, NPs and Mito-NPs, respectively, provided 127- and 74-fold enhancement in DCF fluorescence after 5-min irradiation of 808-nm laser, whereas the pristine DCF solution remained nearly unchanged under the same conditions, indicating the efficient total ROS generation of Mito-NPs. To assess the type-I ROS generation capacities, hydroxyl radical (•OH) and superoxide anion free radical $\left({O_{2}}^{•-}\right)$ were detected, respectively, by HPF and DHR123 probes. As shown in Fig. 2I and fig. S15, the fluorescence signal intensity of HPF increased 67-fold in the NPs solution and 42-fold in the Mito-NPs solution, which might be due to the mitochondrial membrane consuming a part of •OH. The detection of ${O_{2}}^{•-}$ by the DHR123 indicator showed a similar tendency with the presence of NPs and Mito-NPs (Fig. 2J and fig. S16). The electron spinning resonance (ESR) spectra further proved the efficient production of •OH and ${O_{2}}^{•-}$ in Mito-NPs using 5,5-dimethyl-1-pyrroline *N*-oxide (DMPO) under 808-nm laser irradiation (fig. S17). These results demonstrate that Mito-NPs have efficient type-I ROS production upon 808-nm light illumination. In addition, the singlet oxygen (O21), a type-II ROS, was detected by the SOSG probe. The findings demonstrated that the O21 production of Mito-NPs was efficient, and their capability was unaffected by the biomimetic coating of mitochondrial membrane (Fig. 2K and fig. S18). Thus, the Mito-NPs enabled an efficient generation of •OH, ${O_{2}}^{•-}$, and O21 through type-I and type-II processes. Moreover, the generation of H_2_O_2_ during light irradiation was verified using the H_2_O_2_ probe (fig. S19). The photothermal performance of Mito-NPs was further explored under 808-nm laser irradiation. Within 10-min irradiation of 808-nm laser, the temperature of the Mito-NPs solution with a concentration of 50 μg ml^−1^ was distinctly increased from 26° to 78°C, and the PTT effect of Mito-NPs exhibited a concentration-dependent behavior (Fig. 2, L and M). Power-dependent temperature variation was also demonstrated (fig. S20). Based on the photothermal measurements, the η of Mito-NPs was determined to be ~80.3% according to the previously reported method (fig. S21), which is comparable to recently reported PTT agents. The Mito-NPs have similar photothermal performances with pristine NPs without mitochondria biomimetics (figs. S22 to S24). The superior PTT consistency of Mito-NPs was demonstrated using 10 cycles of consecutive laser on/off examination compared with the Food and Drug Administration–approved dye indocyanine green (Fig. 2N). Notably, compared with the YBSe-4H (small molecule, the monomer of YBSe-SS) NPs and YBSe-S (without flexible carbon chains) NPs, YBSe-SS NPs exhibited higher PCE, which might be the introduction of long alkyl chains enhancing the nonradiative transition rate (figs. S25 and S26 and table S2). Also, the YBSe-SS NPs had a stronger ROS production ability than YBSe-4H NPs and a brighter fluorescence emission than YBSe-S NPs under the same conditions. Theoretical calculations demonstrated the rate constants of radiative transition, nonradiative transition, and intersystem crossing for YBSe-SS in aqueous solution, further indicating its potential photothermal, photodynamic, and fluorescent properties (fig. S27) (*72*). As summarized in Fig. 2O, the Mito-NPs could realize NIR-II fluorescence, type I and type II PDT, and PTT effects upon single excitation of an 808-nm laser, demonstrating the good potential of Mito-NPs for phototherapy.

### Photocatalytic performance of Mito-NPs

**Fig. 3.**
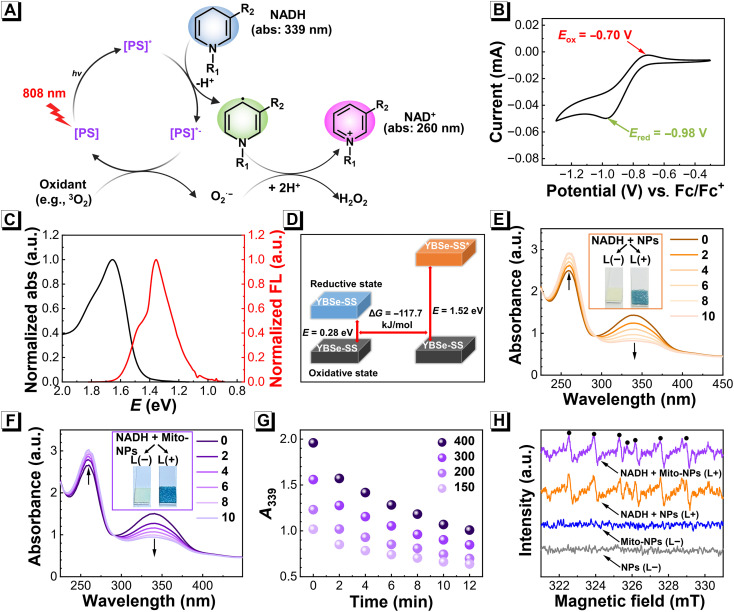
NIR-activated photocatalysis. (**A**) Schematic illustration of the photocatalytic cycle of YBSe-SS PS in NADH oxidation under 808-nm light irradiation. (**B**) Cyclic voltammograms of YBSe-SS as PS in dichloromethane (DCM) with 0.1 M (n-Bu)_4_N^+^PF_6_^−^ as a supporting electrolyte. Fc/Fc^+^ was used as an external reference. (**C**) Absorption and fluorescence spectra of YBSe-SS molecule in DCM. (**D**) Gibbs free energy of ET between the initial state and the excited state of the YBSe-SS molecule in DCM. Photocatalytic oxidation of NADH (240 μM) under 808-nm laser irradiation for 10 min by NPs (**E**) and Mito-NPs (**F**) monitored by absorbance changes. The inset is the chromogenic reaction for H_2_O_2_ detection. (**G**) Concentration and time dependence of photocatalytic reaction of NADH with Mito-NPs (50 μg ml^−1^). (**H**) ESR spectrum of NAD• radicals trapped by 5-(2,2-dimethyl-1,3-propoxycyclo-phosphoryl)-5-methyl-1-pyrroline-*N*-oxide (CYPMPO) with various treatments. a.u., arbitrary unit.

The photocatalytic conversion behavior was investigated on the basis of the excellent physicochemical properties of Mito-NPs under excitation by an NIR light source. In the case of NADH, a vital reductase participating in the maintenance of the intracellular redox balance is very susceptible to oxidation to NAD^+^ [nicotinamide adenine dinucleotide (oxidized form)] (*73*). The possible photocatalytic mechanism for the oxidation of NADH to NAD^+^ during activating the PS (Fig. 3A). When the PS molecule was excited to be an excited state (PS*), a photoinitiated reaction of disproportionation triggered an efficient electron transfer (ET) in the [PS]*, and thus, the NADH was oxidized with a generation of [PS]*^−^, while the ET of [PS]*^−^ could react with the oxidant, such as O23, producing ${O_{2}}^{•-}$, H_2_O_2,_ etc. Next, we carried out practical experiments to understand the mechanism. Cyclic voltammetry (CV) experiments were performed and indicated that the oxidation potential (*E*_ox_) and reduction potential (*E*_red_) of YBSe-SS were, respectively, −0.70 and − 0.98 V (versus Fc/Fc^+^) (Fig. 3B and fig. S28). Combining with the excitation energy of the YBSe-SS in Fig. 3C, the Gibbs free energy (Δ*G*) of photoinitiated ET was investigated using the Rehm-Weller equation (*46*, *74*). The Δ*G* of ET was estimated to be −117.7 kJ mol^−1^, demonstrating the thermodynamic practicability of the intermolecular photoinitiated ET (Fig. 3D). Next, the photocatalytic capacity of NPs and Mito-NPs was represented by monitoring the NADH absorption change. The absorption of the pure NADH was almost unchanged under 808-nm laser illumination for 10 min (fig. S29). Also, no change in absorption was observed in the group of NADH mixing with Mito-NPs (50 μg ml^−1^) without laser irradiation (fig. S30). In contrast, when NADH was mixed with the NPs or Mito-NPs, the special maximum absorption of NADH at 339 and 260 nm was, respectively, decreased and strengthened with the increased time under 808-nm laser irradiation (Fig. 3, E and F). Moreover, there was an obvious generation of H_2_O_2_ in the system after illumination compared to the control group without illumination in an indicator paper. Following that, the concentration-dependent effect of photocatalytic performances of Mito-NPs and NPs was also demonstrated (Fig. 3G and fig. S31). To further validate the effectiveness of photocatalytic performances of Mito-NPs, ESR was used to trap radical intermediates NAD• radical during photocatalytic oxidation of NADH using 5-(2,2-dimethyl-1,3-propoxycyclo-phosphoryl)-5-methyl-1-pyrroline-*N*-oxide (CYPMPO) as the spin trap. With light irradiation, the ESR spectra were found with a carbon-centered NAD• radical in the NPs and Mito-NPs groups (Fig. 3H), which was generated by a photoreaction (*35*). These photoreactions simply do not happen without laser irradiation. Notably, the photocatalytic reaction of NPs or Mito-NPs still decreased under O21-eliminating environment using sodium azide (NaN_3_), demonstrating the feasibility of the photocatalytic reaction of NPs or Mito-NPs in a hypoxic environment (figs. S32 and S33). Therefore, Mito-NPs have demonstrated their effectiveness as the NIR-activated photocatalytic agent, holding good potential in the regulation of intracellular redox balance and hypoxia-overcoming applications.

### Mitochondrial regulation

**Fig. 4.**
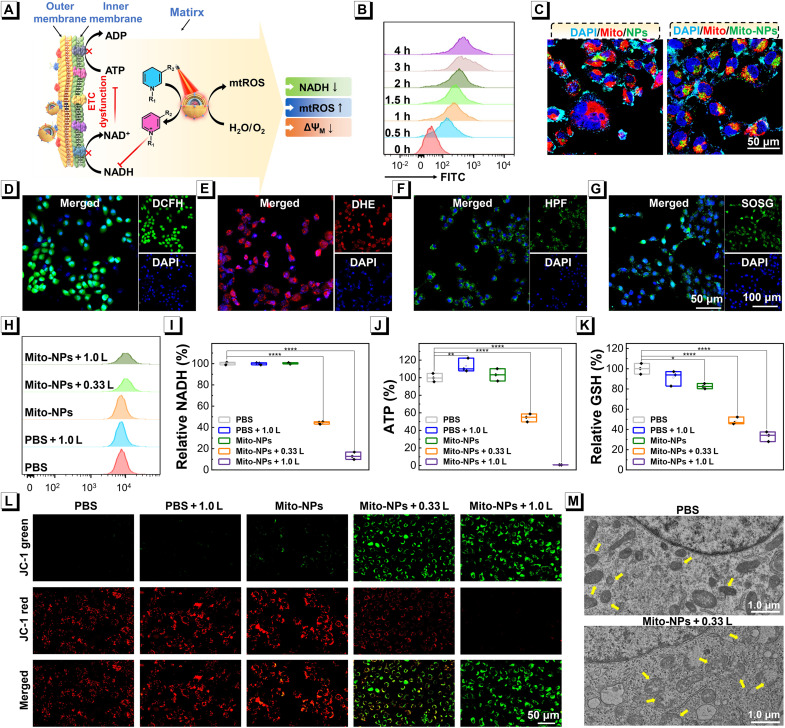
Mitochondrial regulation of Mito-NPs. (**A**) Schematic illustration of the mitochondrial regulation response to Mito-NPs upon irradiation at 808 nm. (**B**) Cell uptake of Mito-NPs (50 μg ml^−1^) by 143B cells at different times via flow cytometry. (**C**) Colocalization images of pristine NPs and Mito-NPs with costaining of Mito-Tracker Red and 4′,6-diamidino-2-phenylindole dihydrochloride (DAPI). Intracellular total ROS generation by DCFH (**D**), •OH by HPF (**E**), ${O_{2}}^{•-}$ by DHE (**F**), and O21 by SOSG (**G**) of Mito-NPs upon light irradiation. (**H**) mtROS change detected via MitoSOX Red mitochondrial superoxide indicator in 143B cells with various treatments. Quantitative analysis of intracellular NADH (**I**), ATP (**J**), and GSH (**K**) levels in 143B cells with different treatments. (**L**) JC-1 staining of 143B cells after various treatments. (**M**) Bio-TEM images of micromorphological changes of mitochondria of 143B cells. Statistical analysis was performed via one-way analysis of variance (ANOVA). *P < 0.05, **P < 0.01, and ****P < 0.0001. Data are presented as means ± SD (*n* = 3).

Taking advantage of the prominent multiple photoactivity, we proposed a hypothesis for the biological effect and anticancer performances of Mito-NPs with efficient photocatalysis in mitochondria (Fig. 4A). To demonstrate this hypothesis, we investigated the cell uptake and intracellular localization of Mito-NPs in human osteosarcoma 143B cells. As shown in Fig. 4B, flow cytometry analysis found that fluorescein isothiocyanate (FITC)–labeled Mito-NPs have efficient cell uptake in 143B cells with a typical time-dependent variation. Moreover, confocal laser scanning microscope (CLSM) imaging was applied to visualize the intracellular localization of FITC-labeled Mito-NPs. The results found that FITC-labeled Mito-NPs showed obvious overlapping with the mitochondria indicator MitoTracker Red. As expected, the mitochondria-targeting capability of Mito-NPs was far beyond that of pristine NPs without mitochondria membrane biomimetic design (Fig. 4C and fig. S34). Furthermore, the result demonstrated that when the camouflaging NPs with homologous tumor cells’ membranes, it did not enhance mitochondrial-targeting efficiency (fig. S35), and the Mito-NPs were barely colocated with mitochondria of normal mouse fibroblast L929 cells (fig. S36). Next, the intracellular ROS generations of Mito-NPs were determined under 808-nm laser irradiation by DCFH-diacetate. As shown in Fig. 4D and fig. S37, with 5 min 808-nm laser irradiation, the cells with Mito-NPs treatment exhibited bright green fluorescence, indicating efficient ROS generation. We also used the corresponding dihydroethidium (DHE), HPF, SOSG indicators, and H_2_O_2_ probe to investigate the intracellular ${O_{2}}^{•-}$, •OH, O21, and H_2_O_2_ produced by photoactivated Mito-NPs (Fig. 4, E to G, and fig. S38). These results found that Mito-NPs activated all three indicators in cells upon 808-nm light irradiation, demonstrating their effectiveness in the •OH, ${O_{2}}^{•-}$, and O21 production. Moreover, the mitochondrial ROS (mtROS) was further investigated via a mtROS probe MitoSOX Red. The flow cytometry analysis found that the Mito-NPs induced efficient mtROS improvement in a dose-dependent manner of 808-nm light irradiation (Fig. 4H). These results demonstrated that mitochondria-targeting Mito-NPs could improve intracellular ROS, especially in mitochondria.

Next, the intracellular photocatalytic oxidation of Mito-NPs was detected using the NAD^+^/NADH Assay Kit with WST-8 for groups with various treatments, including phosphate-buffered saline (PBS), PBS with light irradiation (1.0 W cm^−2^) (PBS + 1.0 L), Mito-NPs, Mito-NPs with light irradiation (0.33 W cm^−2^) (Mito-NPs + 0.33 L), and Mito-NPs with light irradiation (1.0 W cm^−2^) (Mito-NPs + 1.0 L). As shown in Fig. 4I, the intracellular NADH contents were both notably decreased under the treatment of Mito-NPs + 0.33 L and Mito-NPs + 1.0 L. The intracellular NADH content also showed a decreased tendency with the concentration of Mito-NPs increasing (fig. S39). These results indicated that Mito-NPs upon laser irradiation induced photocatalytic oxidation of NADH through the PCT effect. The treatment of Mito-NPs + 0.33 L had a slight photocatalytic oxidation of NADH in L929 cells (fig. S40). Subsequently, adenosine triphosphate (ATP) synthesis responds to the NADH/NAD^+^ balance, and mitochondria function was studied in the cells after being treated with Mito-NPs and light irradiation. As shown in Fig. 4J, Mito-NPs + 0.33 L and Mito-NPs + 1.0 L induced ~40 and 100% decrease in intracellular ATP content, respectively. These results suggested that the Mito-NPs with PCT effect could effectively disturb the electron transport chain process of mitochondria. Also, under 808-nm light irradiation, the glutathione (GSH) level of cells was decreased by Mito-NPs (Fig. 4K). To further determine the multiple photoactivity on the structure of mitochondria, mitochondrial membrane potential (MMP; ΔΨ_M_) was detected using a 5,5,6,6-tetrachloro-1,1,3,3-tetraethylbenzimidazolylcarbocyanine iodide (JC-1). When the carbonyl cyanide 3-chlorophenylhydrazone (CCCP; the standard positive control) and CCCP + 1.0 laser (L) exhibited typical JC-1 fluorescence transformation between red and green, the PBS, PBS + 1.0 L, and Mito-NPs groups barely induced the transformation of JC-1 fluorescence (Fig. 4L and fig. S41). However, 143B cells treated with Mito-NPs + 0.33 L and Mito-NPs + 1.0 L presented evident green fluorescence, suggesting a large MMP depolarisation. Furthermore, the superiority of Mito-NPs on targeting mitochondria was proved via JC-1 indicator, intracellular ATP, and NADH contents compared with pristine NPs (figs. S42 to S44). The morphological changes of mitochondria in 143B cells were observed using biological TEM (Bio-TEM). From Fig. 4M, compared with the PBS group, the mitochondrial structure in the Mito-NPs + 0.33 L group was seriously damaged, as follows from the increased membrane density, reduced or absent mitochondrial cristae, mitochondrial rupturing, and cavitation. The above results indicate that the Mito-NPs could regulate the physiological functions of the mitochondria through PDT and PCT effects.

### In vitro anticancer performances

**Fig. 5.**
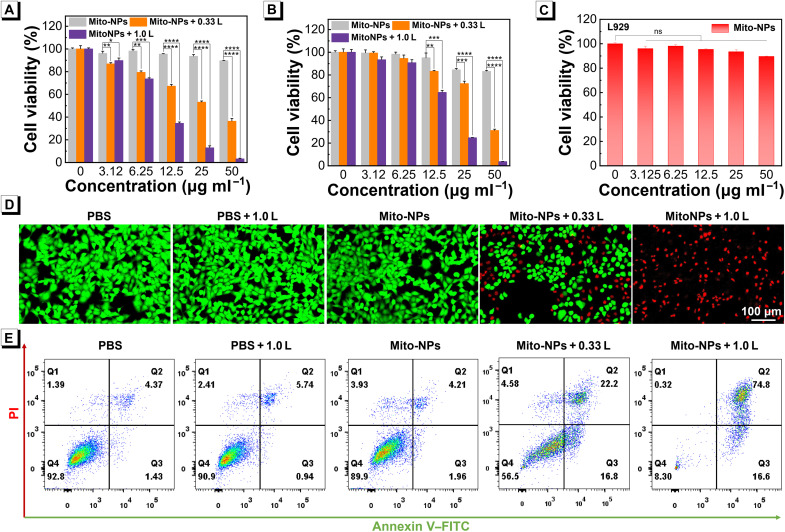
In vitro phototherapy performances of Mito-NPs. Cell viability of 143B (**A**) and K7M2 (**B**) cells after being treated with Mito-NPs and 8-min irradiation of 808-nm laser (0.33 and 1.0 W cm^−2^). (**C**) Cell viability of L929 cells coincubating with Mito-NPs for 24 hours. (**D**) Fluorescence images of 143B cells stained with calcein-AM (green) and propidium iodide (PI) (red) upon different treatments. (**E**) Flow cytometry plot of apoptotic state in 143B cells after various treatments. Statistical analysis was performed via one-way ANOVA. *P < 0.05, **P < 0.01, ***P < 0.001, and ****P < 0.0001. ns, no significance. Data are presented as means ± SD (*n* = 3).

The in vitro biocompatibility and anticancer activities of Mito-NPs upon irradiation at 808 nm were independently studied. Using the Cell Counting Kit-8 (CCK-8) assay, the biocompatibility of the Mito-NPs was found with negligible cytotoxicity toward 143B cells, murine osteosarcoma K7M2-WT (K7M2), and normal mouse fibroblast cells (L929) after 24-hour incubation (Fig. 5, A to C). However, upon light irradiation at 808 nm, the same concentrations of Mito-NPs showed obvious cancer cell killing against 143B and K7M2 cells, and less than 10% of cell survival was demonstrated in the two cells with Mito-NPs treatment (50 μg ml^−1^). Notably, Mito-NPs showed obvious cancer cell killing against osteosarcoma cells upon 808-nm light irradiation (0.33 W cm^−2^ ). These results found that efficient PDT and PCT of Mito-NPs upon low-power irradiation of 808-nm light achieved sufficient antitumor performance, with a negligible PTT performance. Furthermore, the pristine NPs with nonmitochondrial targeting showed lower cytotoxicity toward 143B cells under the same concentration, which confirms the advantages of mitochondrial targeting (fig. S45). Using CLSM imaging, live/dead cell staining of tumor cells treated with Mito-NPs upon irradiation at 808 nm was further investigated. When negligible cell killing was found in light irradiation only and Mito-NPs alone, the groups of Mito-NPs + 0.33 L and Mito-NPs + 1.0 L showed notable cell death (Fig. 5D). The cell killing of Mito-NPs + 0.33 L in hypoxic environment showed a slight decrease compared with that in normoxic environment, which indicated that the type I ROS with the photocatalytic process of NADH might play an efficient role in the tumor killing process (fig. S46). It is important that Mito-NPs + 1.0 L caused almost complete tumor cell killing upon 808-nm light irradiation. Complementary, the cell apoptosis and death induced by Mito-NPs upon irradiation at 808 nm were verified via flow cytometry (Fig. 5E). These results indicated that the Mito-NPs have an efficient tumor cell photoablation upon 808-nm light irradiation.

### Whole-genome RNA-seq of photoactivation performance

**Fig. 6.**
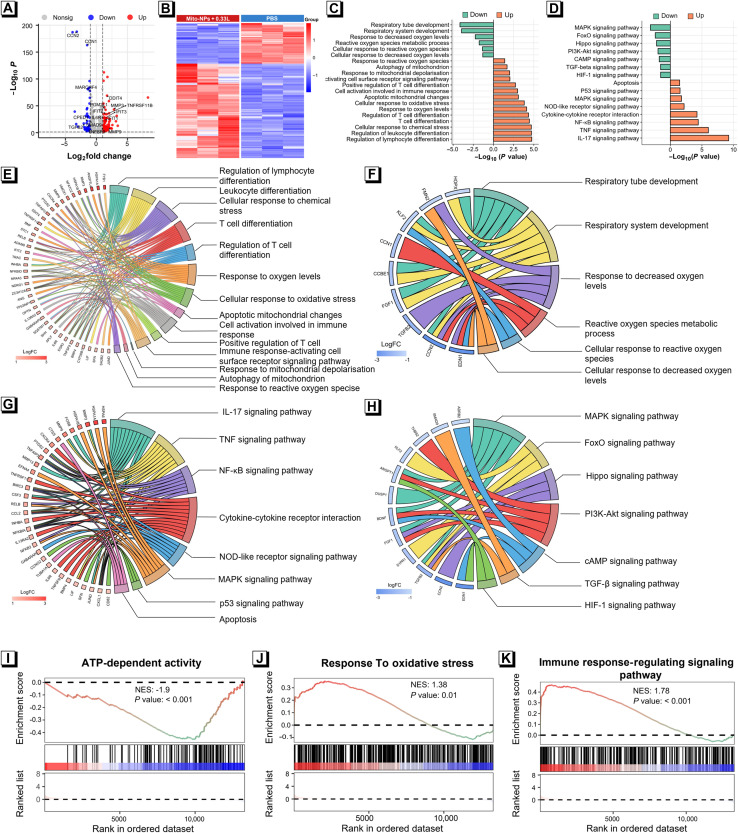
RNA-seq analysis associated with mitochondria and immune pathway. (**A**) Volcano map showing downregulated (blue dots) and upregulated genes (red dots). (**B**) Heatmap illustrating mRNA changes in DEGs after different treatments (*n* = 3). GO (**C**) and KEGG (**D**) enrichment analysis of the DEGs associated with metabolic and immune pathways after different treatments. Chordal plots of GO analysis for up-regulated (**E**) and down-regulated (**F**) DEGs after different treatments. Chordal plots of KEGG analysis for up-regulated (**G**) and down-regulated (**H**) DEGs after different treatments. (**I**) GSEA of DEGs showing gene set enrichment associated with ATP-dependent activity following Mito-NPs + 0.33 L treatment. (**J**) GSEA of DEGs showing gene set enrichment associated with response to oxidative stress activity following Mito-NPs + 0.33 L treatment. (**K**) GSEA of DEGs showing gene set enrichment associated with the immune response following Mito-NPs + 0.33 L treatment.

According to the above experiments, Mito-NPs could induce obvious mitochondria photocatalysis under NIR light irradiation. To further define the biological mechanism of photocatalytic performances, genome-wide RNA sequencing (RNA-seq) was performed on the 143B cells with Mito-NPs + 0.33 L treatment (Fig. 6). The principal components analysis (PCA) indicated the clear separation between groups and high consistency within groups but high-level differences between the two groups (fig. S47). Among the transcriptional profile of 61,806 genes, 3725 differentially expressed genes (DEGs) (|log_2_(fold change)| ≥1.0, *P* value < 0.05) were identified between the Mito-NPs and the control PBS groups. We extracted the mitochondrion and immune response relative genes from the DEGs for further analysis in detail. As displayed in Fig. 6 (A and B), cellular morphology– and metabolism-associated genes—including CCN1, CCN2, MARCHF4, CPED1, ATP1B1, SAMD9, and TGFB2—were obviously down-regulated in the cells with Mito-NPs + 0.33 L treatment. Moreover, genes associated with apoptosis and autophagy—such as DDIT4, SQSTM1 and MMPs—were up-regulated. Furthermore, the treatment of Mito-NPs + 0.33 L induced the up-regulation of the nuclear factors of activated T cells (NFAT) family (including NFATC1, NFATC2, and NFATC4), tumor necrosis factor (TNF) family (including TNFRSF11B and TNFSF9), and interleukin (IL) family (such as IL6R), demonstrating the activation of immune responses. These results suggest that the phototherapy of Mito-NPs could induce energy metabolism disorders and typical immune activation. The Gene Ontology (GO) to garner insights into the biological courses, cellular elements, and biofunctionalities is displayed in Fig. 6 (C and E and F), from which the treatment of Mito-NPs + 0.33 L mainly influenced negative regulation of mitochondrial function, including the respiratory system, cellular response to ROS, and cellular response to decreased O_2_ levels. Conversely, the apoptosis and autophagy triggered by mitochondrial damage and immune activities such as T cell differentiation and lymphocyte differentiation were regulated positively. From the Kyoto Encyclopedia of Genes and Genomes (KEGG) analysis (Fig. 6, D and G and H), there were notable differences in the pathways of apoptosis regulation, mitochondria, and immune activation. For instance, the metabolism-related pathways, including mitogen-activated protein kinase, FoxO, and Hippo, especially phosphatidylinositol 3-kinase (PI3K)–Akt related to NADH regulation, were down-regulated. Western blot analysis also proved that the PI3K-Akt protein expressions were down-regulated with an obvious up-regulation of their phosphorylation after being treated with Mito-NPs + 0.33 L, which is consistent with the RNA-seq analysis (fig. S48), and immune-related pathways were positively regulated. Gene set enrichment analysis (GSEA) showed that ATP-dependent activity was down-regulated under Mito-NPs + 0.33 L treatment, which is consistent with depleted cellular NADH levels (Fig. 6, I to K). The cellular response to oxidative stress–related genes was distinctly up-regulated to resist photodynamic and photocatalytic processes. Furthermore, the up-regulation of the immune-response signaling pathway was and consistent with the GO and KEGG analyses. These results offer genomics evidence for the proof that Mito-NP–mediated phototherapy affected the energy metabolism in the mitochondria and activated the immune response.

### In vivo NIR-II fluorescence imaging

**Fig. 7.**
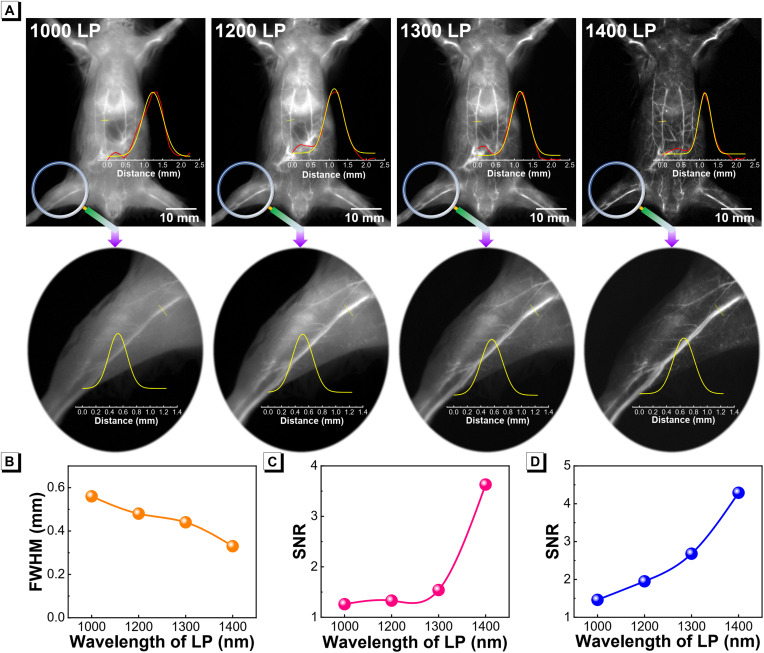
NIR-II angiography. (**A**) In vivo NIR-II angiography of the blood vessels of the whole body and inner thigh using Mito-NPs labeling after different LP filters. (**B**) Corresponding FWHM analysis of yellow-marked vessels of the whole body. SNR analysis of the marked vessels in the whole body (**C**) and inner thigh (**D**). Excitation source: 808-nm laser.

To validate the feasibility of Mito-NPs for in vivo NIR-II angiography, the experiment of whole-body blood vessel imaging was conducted via intravenous injection of Mito-NPs and then dynamically visualized in an NIR-II imager with an 808-nm excitation. The whole-body vessels can be recognized with 1-min postinjection of Mito-NPs, and the NIR-II fluorescence signals were recorded after various long-pass (LP) filters in the 1000- to 1400-nm range. As shown in Fig. 7A, it is worth noting that the image of 1400-nm LP has a higher resolution in the whole-body angiography compared with those of 1000- to 1300-nm LP. To further demonstrate the advantages of NIR-II angiography, the main blood vessels on the body and the inner thigh were selected, respectively, to analyze their full width at half maximum (FWHM) and signal-to-noise ratio (SNR). For the body blood vessel, the FWHM values of the yellow-marked vessel in NIR-II images raised along with the increase of filter wavelength, and the NIR-II images under the 1400 LP filter achieved the clearest vessel profile (Fig. 7B). The SNR analysis of the yellow-marked vessel further confirmed the above results (Fig. 7C). Moreover, zooming on the vessel of the inner thigh was further performed and found that the NIR-II images on the vessel of the inner thigh under the 1400 LP filter provided the highest SNR of 4.29, which is 2.8-fold higher than those of the 1000 LP filter (Fig. 7D). These results demonstrated the superiority of Mito-NPs for high-resolution NIR-II angiography, especially beyond 1400 nm. With these advantages, the in vivo delivery of Mito-NPs could be visualized by NIR-II imaging.

### In vivo phototheranostics of primary tumor

**Fig. 8.**
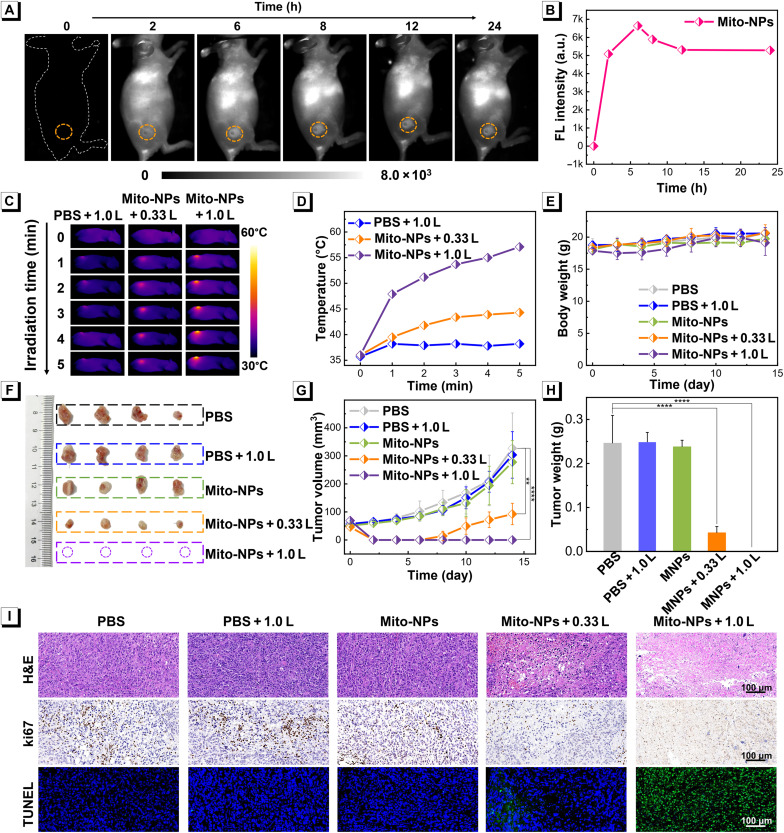
In vivo imaging-guided therapy. (**A**) In vivo NIR-II fluorescence imaging of tumor targeting of Mito-NPs. (**B**) Fluorescence intensities of tumor site at different postinjection times. (**C**) Infrared thermal images of the mice with 808-nm laser irradiation within 5 min. (**D**) Temperature profiles of tumor as a function of irradiation time. (**E**) Body weight changes of the treated mice within 14 days of treatment. (**F**) Digital images of tumors were separated from the treated mice at the end of the treatments. (**G**) Tumor growth curves of different treatment groups within 14 days of treatments. (**H**) Tumor weight analysis of the treated mice after different treatments. (**I**) Representative H&E, ki67, and TUNEL staining of tumor tissue after different treatments. Statistical analysis was performed via one-way ANOVA. **P < 0.01 and ****P < 0.0001. Data are presented as means ± SD (*n* = 4).

To evaluate in vivo phototheranostics of the primary tumor, osteosarcoma 143B tumor-bearing nude mice were established, and the tumor volume of nearly 50 to 80 mm^3^ was chosen for the next imaging application. For tumor-targeted NIR-II fluorescence imaging, the mice were treated with Mito-NPs (100 μl, 1.0 mg ml^−1^) via intravenous injection and then placed in a NIR-II fluorescence imager for real-time observation. As shown in Fig. 8 (A and B), the NIR-II fluorescence signal at tumor sites gradually increased within 6 hours postinjection and reached a maximum at 6 hours postinjection, suggesting their relatively efficient tumor location of Mito-NPs. The tissue biodistribution of Mito-NPs also found the efficient tumor retention of Mito-NPs (fig. S49). Because of their efficiency in tumor targeting, we thus further investigated the in vivo photothermal heating performance of Mito-NPs in tumor lesions. After intravenous injection of Mito-NPs (100 μl, 1.0 mg ml^−1^), the treated mice were irradiated with an 808-nm laser at the intensity of 0.33 or 1.0 W cm^−2^, and then the temperatures at tumor sites were monitored by an infrared thermal imaging camera. As depicted in Fig. 8 (C and D) and fig. S50, after 5-min irradiation, the tumor temperatures of the mice treated with 0.33 and 1.0 W cm^−2^ were increased to 44.3° and 57.1°C, respectively. In contrast, the tumor in PBS-treated mice showed a slight temperature change after 5 min of light radiation of 808-nm laser at 1.0 W cm^−2^. In addition, the Mito-NPs with three times injection still showed good tumor elimination, indicating no risk of drug resistance (fig. S51). These results demonstrated good in vivo photothermal heating performance of Mito-NPs, which is consistent with sufficient tumor targeting of Mito-NPs.

Taking advantage of tumor targeting in vivo photothermal heating performances, the in vivo phototherapy of the primary tumor was evaluated in 143B tumor-bearing nude mice. The mice with a tumor volume of ~50 to 80 mm^3^ were randomly divided into five groups (*n* = 4 for each group): PBS, PBS with light (1.0 W cm^−2^) (PBS + 1.0 L), Mito-NPs, Mito-NPs with light (0.33 W cm^−2^) (Mito-NPs + 0.33 L), and Mito-NPs with light (1.0 W cm^−2^) (Mito-NPs + 1.0 L). The PBS or Mito-NPs were intravenously injected into mice, followed by 808-nm laser irradiation (0.33 or 1.0 W cm^−2^) for 10 min at 6 hours postinjection. There is no obvious difference among these five groups in mouse weights within the 14 days of treatment (Fig. 8E). As shown in Fig. 8 (F and G), the tumor in PBS + 1.0 L and Mito-NPs groups exhibited a similar growth tendency to the PBS group. In contrast, both Mito-NPs + 0.33 L and Mito-NPs + 1.0 L provided obvious tumor inhibition within 6 days of posttreatment. However, the treatment of Mito-NPs + 0.33 L had typical tumor recurrence after 6 days of posttreatment, and the recurrent tumors were living and growing, whereas Mito-NPs + 1.0 L almost exclusively eliminated tumor without any recurrence within 14 days of treatment. The tumor weight analysis of the mice after 14 days of treatment further confirmed the above results (Fig. 8H). These results demonstrate that Mito-NPs would completely ablate the primary tumor without any recurrence via versatile phototherapeutic performances. To further demonstrate the effectiveness of Mito-NPs with 808-nm light irradiation, tumor biopsy including hematoxylin and eosin (H&E) staining, immunohistochemical staining with the nuclear-associatedantigenki-67 (ki67) marker (proliferating cell marker), and terminal deoxynucleotidyl transferase–mediated deoxyuridine triphosphate nick end labeling (TUNEL) assay, was applied. As shown in Fig. 8I, the efficient apoptosis of tumor cells was achieved in the group of Mito-NPs + 1.0 L, as evidenced by the highly fragmentized nucleus, the absence of the ki67 marker, and the intense TUNEL signal, indicating the antitumor efficiency of Mito-NPs with 808-nm light irradiation. According to the International Harmonization of Nomenclature and Diagnostic Criteria for Lesions in Rats and Mice (INHAND), the pathology score based on H&E of tumor tissues was also calculated to confirm the above results (table S3). Furthermore, the efficient production of ROS in the solid tumor was demonstrated in Mito-NPs + 0.33 L (fig. S52). Also, the decreased contents of NADH, ATP, and mitochondrial activity in the solid tumor further investigated and indicated the light-induced mitochondrial damage (figs. S53 to S55).

The preliminary biocompatibility of Mito-NPs treatment and 808-nm light irradiation was further demonstrated via tissue biopsy of H&E staining in the main organs and blood biochemistry analysis after 14 days of treatments (figs. S56 and S57). Besides, the Mito-NPs were efficiently cleaned from the main organs at 72 hours postinjection (fig. S58), and the hemolytic test further demonstrated the good biosafety of Mito-NPs (fig. S59). Thus, these results proved the antitumor effectiveness and good biocompatibility of Mito-NPs.

### Tumor metastasis inhibition of Mito-NPs

**Fig. 9.**
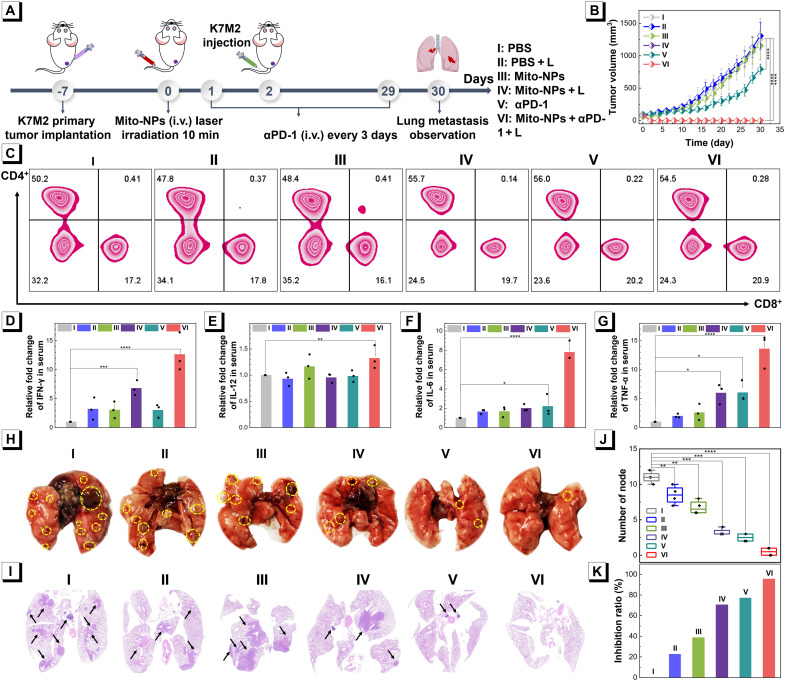
Photoimmunotherapy of metastatic tumor. (**A**) Schematic diagram of the treatment schedule for antimetastasis tumor treatment. (**B**) Tumor growth curves of the primary tumor with different treatments within 30 days of treatment. (**C**) Flow cytometry analysis of CD8^+^ T cells (CD3^+^CD8^+^) in the lymph nodes of the treated mice after different treatments. ELISA assay of IFN-γ (**D**), IL-12 (**E**), IL-6 (**F**), and TNF-α (**G**) in the serum of the treated mice after different treatments. (**H**) Digital photographs of lung tissues from the mice with different treatments. (**I**) H&E staining of the lung after various treatments. (**J**) Quantitative number of nodes in the lung after various treatments. (**K**) Inhibition ratio analysis in various groups. Statistical analysis was performed via one-way ANOVA. *P < 0.05, **P < 0.01, ***P < 0.001, and ****P < 0.0001. Data are presented as means ± SD (*n* = 4).

Encouraged by the decent immune responses and therapeutic effect, we further evaluated lung metastasis inhibition via Mito-NPs photoimmunotherapy combined with immune checkpoint blockade (ICB) with anti-programmed death-1 (αPD-1) antibody therapy (Fig. 9A). The primary osteosarcoma tumors of ~50 to 80 mm^3^ were treated with Mito-NPs + L to induce in situ photoimmunotherapy, and then mouse osteosarcoma K7M2 cells were intravenously injected into immunocompetent Balb/c mice to establish a mouse model for mouse osteosarcoma cancer spontaneous lung metastasis. To combine with ICB therapy, an antibody of αPD-1 was administered every 3 days. The tendency of temperature and growth of primary tumor in Mito-NPs + L groups was consistent with the previous result, and αPD-1 alone also exhibited a mild antitumor performance (Fig. 9B and fig. S60). The body weight of mice with various treatments had no clear differences, indicating good biosafety (fig. S61). To explore the immunotherapy progression, immune cells in lymph nodes were further analyzed via flow cytometry after various treatments. As shown in Fig. 9C and fig. S62, compared with the PBS group, the single treatment of Mito-NPs + L exhibited obvious improvement in the CD8^+^ cytotoxic T lymphocytes in lymphoid tissue and spleen. Also, the Mito-NPs + L + αPD-1 group exhibited high CD8^+^ cytotoxic T lymphocytes of 20.9%, indicating the effectiveness of Mito-NP–induced photoimmunotherapy combined with ICB of αPD-1 in the activation of CD8^+^ cytotoxic T lymphocytes. In addition, the in vitro dendritic cell (DC) maturation was also proved (fig. S63). Moreover, the serum levels of relative cytokines in the treated mice were further investigated by the enzyme-linked immunosorbent assays (ELISA). As shown in Fig. 9 (D to G), after being treated with Mito-NPs + L or αPD-1, all cytokines and proinflammatory mediators—including interferon-γ (IFN-γ), interleukin-12p70 (IL-12p70), IL-6, and TNF-α—displayed a notable increase. Notably, among these treatments, the Mito-NPs + L + αPD-1 group showed the highest improvement in these indicators. Moreover, a concentration-dependent activation of the immune response in Mito-NPs + L was found via cytokine detection (fig. S64). These results indicate that Mito-NP–induced photoimmunotherapy combined with ICB treatment can effectively trigger the systemic immune response.

Lung metastasis of osteosarcoma was recorded after various treatments within 30 days of posttreatment, and the results are shown in Fig. 9 (H and I). The digital and H&E staining photographs of the lung tissue after various treatments showed that the lung metastatic nodes in mice with different treatments, especially combined treatment, were remarkably suppressed compared with the PBS group. The amount and inhibition efficiency of lung metastatic nodes in different mice were statistically analyzed. As shown in Fig. 9 (J and K), the monotherapy of Mito-NPs + L or αPD-1 antibody provided efficient suppression in osteosarcoma cancer spontaneous lung metastasis. However, the metastatic nodes in the combination therapy of Mito-NPs + L + αPD-1 were almost completely inhibited. The amplifying activation of innate immune response may benefit from the multiple union of photoimmunotherapy and ICB effect. In total, via the photoimmunotherapy of Mito-NPs combined with the ICB effect, Mito-NPs activated and recruited immune cells to initiate a good innate immune response for suppressing osteosarcoma cancer spontaneous lung metastasis.

## DISCUSSION

In summary, we reported a biomimetic organic semiconductor photocatalyst for mitochondria-targeted NIR-activatable photocatalytic immunotherapy of protopathic, recurrent, and metastatic tumor. The NIR-responsive conjugated polymer YBSe-SS was designed and further prepared into a biomimetic photocatalyst Mito-NPs. The results indicated that Mito-NPs encapsulated by a mitochondrial membrane achieved an efficient mitochondrial location in tumoral cells. Upon irradiation by 808-nm light, the Mito-NPs exhibited multiple NIR-II activities of PTT, PDT, and fluorescence emission at an impressive efficiency. Notably, effective photocatalytic oxidation of NADH was further realized by the Mito-NPs, and thus, the mitochondrial dysfunction of redox balance and respiratory chain was successfully regulated. With function genomics analysis, the mitochondria-targeted photocatalytic Mito-NPs were found to efficiently regulate the mitochondria function and trigger the immune response under 808-nm light excitation. In vivo experiments showed that the photocatalytic Mito-NPs had an efficient NIR-II fluorescence imaging-guided antitumor function and also achieved photocatalytic immunotherapy for suppressing the recurrence and pulmonary metastasis of osteosarcoma by the combination of ICB. This study developed a promising design toward NIR photocatalytic materials, offering a previously unidentified paradigm for PCT. Although the bionic nanomedicine shows many advantages, large-scale nanotechnology, such as the microfluidic-electroporation continuous platform, should be further developed. Batch-to-batch reproducibility is another issue that deserves consideration for clinical developments.

## MATERIALS AND METHODS

### Preparation of Mito-NPs

First, YBSe-SS NPs (NPs) were prepared through a classic nanoprecipitation method with DSPE-PEG as a surfactant. In brief, 0.25 mg of YBSe-SS molecular powder was mixed with 2.5 mg in 1 ml of THF and then dropped into 9 ml of deionized (DI) water under vigorous stirring for 48 hours. After that, the NP solutions were further washed and concentrated by the ultrafiltration method. Next, according to the Mitochondrial Extraction Kit, the mitochondrial membrane was extracted from the 143B cells via a homogenizer. The resulting mitochondrial membrane was quantified by the BCA Protein Assay Kit and then kept at −80°C for the next use. For mitochondria biomimetics, the concentration ratio of NPs and mitochondrial membrane at 15:1 was repeatedly extruded by an Avanti mini extruder for Mito-NPs preparation.

### Mito-NP characterization

The DLS and zeta potential data of the Mito-NPs solutions were performed using a Malvern Nano ZS 90. TEM images were taken on a transmission electron microscope with an acceleration voltage of 120.0 kV. The fluorescence imaging performances of Mito-NPs with various concentrations in vitro were evaluated by the NIR-OPTICS Series III 900/1700 small animal imaging system. The QY of Mito-NPs was measured using dye IR26 (QY = 0.5%) as a reference.

### Photodynamic performances

The total ROS produced by NPs and Mito-NPs under laser irradiation was detected with the activated probe DCFH. Briefly, NPs or Mito-NPs (50 μg ml^−1^) in 2 ml of DI water with a final concentration of DCFH (40 μM) were irradiated with an 808-nm laser (0.33 W cm^−2^). The fluorescence emission spectra of the various solutions were detected with an excitation wavelength of 460 nm, and the characteristic absorption at 525 nm was collected. A blank DCFH solution (40 μM) with corresponding laser irradiation was applied as a control and a reference. Similarly, the productions of •OH, O21, ${O_{2}}^{•-}$, and H_2_O_2_ were detected by HPF, SOSG, DHR123, and ROS Green H_2_O_2_ probe, respectively (5 μM), under the same parameters.

### Cyclic voltammetry curve

Cyclic voltammogram experiments were conducted using a three-electrode system. In this configuration, glassy carbon served as the working electrode, Ag/Ag^+^ was used as the reference electrode, and platinum sheets were used as the counter electrode. Ferrocene (Fc) was used as the external reference. The Fc or YBSe-SS with a concentration of 1.0 mM, mixed with (n-Bu)4 N^+^PF6^−^ (0.1 M), were dissolved in dichloromethane to obtain the supporting electrolytes. The scanning rate during the measurement was set as 100 mV s^−1^.

### Photocatalytic performances

The photocatalytic oxidation capacity of NPs and Mito-NPs was evaluated by monitoring the absorption of solutions at 339 nm (characteristic absorption peak of NADH molecule) with or without the irradiation of an 808-nm laser. Meanwhile, the production of H_2_O_2_ was detected by Quantofix peroxide test sticks, which would turn from white to blue when exposed to H_2_O_2_. Furthermore, CYPMPO (1 mM) was used as a spin-trap agent for NAD• radicals in solution containing NPs or Mito-NPs and NADH under 808-nm irradiation in vitro.

### Intracellular photodynamic and photocatalytic performances

The cell colocalization analysis among Mito-NPs and mitochondria was detected by CLSM via incubating 143B cells and fluorescein-labeled Mito-NPs. Furthermore, the uptake of fluorescein-labeled Mito-NPs over time was detected by flow cytometry. Various types of ROS produced by Mito-NPs were measured using the corresponding probes, including DCFH, DHE, HPF, and SOSG, and MitoSOX Red probe was used to detect the mitochondrial ${O_{2}}^{•-}$ by flow cytometry. The contents of some key regulatory intracellular molecules—such as NADH, ATP, and GSH—in various groups (PBS, PBS + 1.0 L, Mito-NPs, Mito-NPs + 0.33 L, and Mito-NPs + 1.0 L) were detected using the commercial kits, including NAD^+^/NADH Assay Kit, ATP Assay Kit, and GSH Assay Kit, respectively. The mitochondrial membrane potential was determined by JC-1 dye using CCCP as a positive control. The morphological changes of mitochondria in 143B cells with Mito-NPs + 0.33 L treatment were observed using biological TEM, using PBS treatment as a control.

### Ethical statement

All female mice were obtained from GemPharmatech Co. Ltd. (Nanjing, China). All animal experiments were performed under the guidelines, evaluated, and approved by the ethics committee of Soochow University (SUDA20241210A03).

### Whole-body NIR-II fluorescence imaging

The Balb/c mouse with a body weight of ~20 g was used for whole-body fluorescence angiography. After the abdomen-depilated treatment, Mito-NPs (100 μl, 1.0 mg ml^−1^) were injected into the mouse intravenously, and then the experimental mouse was excited by an 808-nm laser. The NIR-II fluorescence images with different LP filters (1000 to 1400 nm) were collected by a small animal imaging system of NIR-OPTICS Series III 900/1700.

### NIR-II imaging-guided tumor phototherapy

All Balb/c female nude mice (6 to 8 weeks of age) with body weights of ~20 g were used for imaging-guided phototherapy of cancer. 143B cells (100 μl, 2 × 10^7^ cells ml^−1^) were injected subcutaneously into the right lower extremity to establish a tumor model. When the tumor size reached 50 to 80 mm^3^, the experiments of NIR-II imaging and tumor phototherapy were performed, successively. For in vivo fluorescence imaging, the 143B tumor-bearing mice were intravenously injected with 100 μl of NPs (1.0 mg ml^−1^) and 100 μl of Mito-NPs (1.0 mg ml^−1^) solutions, respectively, and then the fluorescence images of tumor sites were detected at various postinjection times (0, 2, 6, 8, 12, and 24 hours) by the NIR-OPTICS Series III 900/1700 small animal imaging system. For tumor treatments, the 143B tumor-bearing mice were randomly divided into five groups of four mice each, including PBS, PBS + 1.0 L, Mito-NPs, Mito-NPs + 0.33 L, and Mito-NPs + 1.0 L. According to the various experimental conditions, mice were intravenously injected with PBS (100 μl) or Mito-NPs aqueous solution (100 μl, 1.0 mg ml^−1^) at 6 hours postinjection (based on the result of fluorescence imaging). The tumor sites of mice in the Mito-NPs + 0.33 L and Mito-NPs + 1.0 L groups were irradiated with an 808-nm laser for 10 min. During the irradiation process, temperature changes of the tumor sites were taken as a photographic record with the Ti480 thermal imaging camera. After the phototherapy, all mice’s body weights and tumor volumes were monitored every 2 days during the following 14 days (the day they received laser treatment was set as day 0). At the end of the therapeutic process, all of the mice were euthanized following animal ethics guidelines. The tumors from the various groups were collected and immediately fixed in a 4% formaldehyde solution to evaluate the antitumor effect. Then, the tumor tissue was sectioned into 10-μm slices by a histotome. Furthermore, sectioned tissue slides of solid tumors were stained for H&E, ki67, and TUNEL staining.

### Photoimmunotherapy of metastatic tumor

The K7M2 tumor-bearing Balb/c female mice with a tumor at 50 to 80 mm^3^ volume were randomly divided into six groups of four mice each, including PBS, PBS + L, Mito-NPs, Mito-NPs + L, αPD-1, and Mito-NPs + L + αPD-1 groups. Then, different phototherapies or immunotherapy was given respectively. The αPD-1 and Mito-NPs + L + αPD-1 group were given αPD-1 solution (0.5 mg ml^−1^, 100 μl) every 3 days from the eight day to the end of treatment. Different groups of mice were all given K7M2 cell suspension (5 × 10^6^) through intravenous injection on the 9th day. The tumor and lung tissue of mice were collected for subsequent analysis after 30 days. At 24 hours posttreatment, the draining inguinal lymph nodes were peeled off and filtrated by a cell strainer (a size of 70 μm), the resulting samples were treated with the LIVE/DEAD Fixable Dead Cell Stain Kit (1:200) at 4°C for 20 min. After being blocked with mouse serum for 10 min, the samples were, respectively, stained with CD3-FITC (1:100), CD4–phycoerythrin (PE)/Cyanine7 (1:100), CD8a–allophycocyanin (APC)/Cyanine7 (1:100), CD80-PE (1:100), and CD86-APC (1:100) antibodies at 4°C for 30 min. For flow cytometry analysis, the cell samples with PBS washing were resuspended in the staining buffer. The blood collected from the above mice was left for 1 hour and centrifuged for 10 min at 2000 rpm to receive serum. The serum concentrations of various cytokines—such as TNF-α, IFN-γ, IL-6, and IL-12—were analyzed with the corresponding ELISA kits according to the manufacturer’s protocols. For the lung metastases at 30 days posttreatment, the lung tissues from the mice with various treatments were observed, and the numbers of metastatic nodules were recorded to evaluate the lung metastasis inhibition of each group. The tissue biopsy of H&E staining was applied for the histology assessment.

### Statistical analysis

The data used for statistical analysis were at least three parallel replicates. The means ± SD was used to present the data. Besides, statistical analysis was performed with a one-way analysis of variance (ANOVA) test. Statistical significance was defined as ****P < 0.0001, ***P < 0.001, **P < 0.01, and *P < 0.05, and n.s. represents no significance.

## Supplementary Materials

### This PDF file includes:

Supplementary Text Figs. S1 to S64 Tables S1 to S3 References
