# Supplementary material for: Mitochondria-localized photocatalyst of biomimetic organic semiconductor nanoparticles for NIR-activatable photocatalytic immunotherapy
Source: Sci Adv. 2025 Dec 5;11(49):eadx4850. doi: 10.1126/sciadv.adx4850 (PMC12680062; doi:10.1126/sciadv.adx4850)
Supplement: Supplementary file 1 — Supplementary Text Figs. S1 to S64 Tables S1 to S3 References [file sciadv.adx4850_sm.pdf]

Science Advances
AAAS

Supplementary Materials for

Mitochondria-localized photocatalyst of biomimetic organic semiconductor nanoparticles for NIR-activatable photocatalytic immunotherapy

Yu Wang *et al.*

Corresponding author: Shengliang Li, lishengliang@suda.edu.cn; Yuanfeng Chen, chenyuanfeng@gdph.org.cn

Sci. Adv. **11**, eadx4850 (2025)
DOI: 10.1126/sciadv.adx4850

This PDF file includes:

Supplementary Text
Figs. S1 to S64
Tables S1 to S3
References

Supplementary Text

Materials and Methods

All chemical reagents for organic synthesis were obtained from commercial suppliers (Derthon Optoelectronics Materials Science Technology Co., Ltd, InnoChem, TCI) and used without further treatment. β-nicotinamide adenine dinucleotide and reduced disodium salt (β-NADH) and 5,5-Dimethyl-1-pyrroline N-oxide (DMPO) were purchased from Sigma-Aldrich. 5-(2,2-dimethyl-1,3-propoxycyclophosphoryl)-5-methyl-1-pyrroline N-oxide (CYPMPO) was obtained from MedChemExpress (Shanghai, China). 1,2-Distearoyl-sn-glycero-3-phosphoethanolamine-N-[methoxy(polyethylene glycol)-2000] (DSPE-PEG) and fluorescein-labelled DSPE-PEG were purchased from Yarebio Technology Co., Ltd (Shanghai, China) and Ponsure Biotechnology Company (Shanghai, China), respectively. 1-benzothiopyrylium perchlorate derivative (IR26) and indocyanine green (ICG) were purchased from Exciton (USA) and Shanghai Yuanye Bio-Technology Co., Ltd (Shanghai, China), respectively. 2',7'-Dichlorodihydrofluorescein diacetate (DCFH-DA), hydroxyphenyl fluorescein (HPF), and dihydrorhodamine 123 (DHR123) were obtained from Sigma-Aldrich (Shanghai, China), whereas singlet oxygen sensor green (SOSG) and MitoSOX Red were purchased from ThermoFisher Scientific Co., Ltd (Shanghai, China). ROS Green^{TM} H_2O_2 Probe was purchased from Maokang Bio. (Shanghai, China). Dulbecco's Modified Eagle Medium (DMEM) and the Annexin V-FITC Apoptosis Detection Kit (CA1020) was obtained from Beijing Solarbio Science & Technology Co., Ltd (Beijing, China). Penicillin-streptomycin and fetal bovine serum (FBS) were obtained from Pricella Life Science&Technology Co., Ltd. Phosphate buffer saline (PBS) was purchased from Servicebio Technology Co., Ltd (Wuhan, China). Pancreatin was obtained from Anhui KETU Biotechnology Co., Ltd. 3-(4,5-Dimethylthiazol-2-yl)-2,5-diphenyltetrazolium bromide (MTT) was obtained from Keqing Biotechnology Co., Ltd (Suzhou, China). 4',6-Diamidino-2-phenylindole (DAPI), Reactive Oxygen Species Assay Kit including DCFH and DHE probes, Calcein-AM, propidium iodide (PI), Mitochondrial Extraction Kit, and Micro Reduced Glutathione (GSH) Assay Kit were provided by Beijing Solarbio Science & Technology Co., Ltd (Beijing, China). LysoTracker™ Deep Red and MitoTracker™ Deep Red were purchased from ThermoFisher Scientific Co., Ltd (Shanghai, China). NAD^+/NADH Assay Kit with WST-8, ATP Assay Kit, Mitochondrial Membrane Potential Assay Kit with JC-1 (JC-1), Apoptosis and Necrosis Assay Kit, and BCA Protein Assay Kit and Annexin V-FITC Apoptosis Detection Kit were purchased from Beyotime Biotech. Inc. (Shanghai,

China). ELISA kits for TNF-α, IFN-γ, IL-6 and IL-12 assay were all obtained from Aimeng youning (Shanghai, China). FITC anti-mouse CD3 (catalogue: 100204), PE/Cyanine7 anti-mouse CD4 (catalogue: 116016), APC/Cyanine7 anti-mouse CD8a (catalogue: 100714), PE anti-mouse CD80 (catalogue: 104708) and APC anti-mouse CD86 (catalogue: 105012) antibodies were obtained from BioLegend, Inc. (San Diego, USA). Pan-Akt antibody (A18675), Phospho-Akt antibody (AP0637), PI3K antibody (A27717), and Phospho-PI3K antibody (AP1487) were purchased from ABclonal Biotechnology Co., Ltd. Anti-GAPDH antibody (#2118, 14C10) were purchased from Cell Signaling Technology.

^{1}H NMR and ^{13}C NMR spectra were tested *via* a Bruker Advance III (400 MHz) instrument using Chloroform-D ($CDCl_3$) as the solvent. High-resolution mass spectra (HRMS) were measured *via* a Bruker Ultraflextreme MALDITOF/TOF high-resolution electrospray ionization. A Nano ZS90 (Malvern, UK) was utilized to detect the size distributions and zeta potential. Transmission electron microscopy (TEM) images and element mapping were recorded *via* a transmission electron microscope (TALOS F200X, USA). Electron spin resonance spectrometry (ESR, JES-X320, Japan) was used to determine the production of free radicals. Fluorescence spectra of the YBSe-SS molecules in tetrahydrofuran (THF) solution and nanoparticles were detected by Fluorescence Spectrometer (NIR-VIS, FL3, HORIBA Scientific, USA). The fluorescence intensity changes of DCF, HPF, SOSG and DHR123 in the reactive oxygen species (ROS) detection experiment were recorded by a Fluorescence Spectrometer (PL-TCSPC, HORIBA Scientific, USA). Changchun New Industries Optoelectronics Tech. Co., Ltd (Changchun, China) provided the laser machines (808 nm). The temperature variations were recorded with a thermal imager (Fluke Ti400, IR Fusion Technology, USA). The fluorescence images of cells in the intracellular ROS detection, colocalization, and JC-1 experiments were taken by a Nikon A1R HD25 laser scanning confocal microscope (CLSM, Nikon, Japan). Fluorescence images of live/dead cells were recorded by Zeiss LSM 710 (Carl Zeiss, Germany). Apoptosis and necrosis analysis were recorded by flow cytometry (FCM, FACS Aria III, Becton, Dickinson and Company, USA). The hypoxic environment was obtained by HypoxiaIncubator (billups-rothenberg, USA). NIR-II fluorescence imaging was collected with a small animal imaging system of NIR-OPTICS Series III 900/1700 (Artemis Intelligent Imaging Co., Ltd., China).

Synthesis and characterization of YBSe-SS molecule

To a 50 mL two-neck flask, compound TPBSe (123 mg, 0.1 mmol), IC (109 mg, 0.4 mmol), Pyridine (0.25 mL), and $CHCl_3$ (15 mL) were carefully degassed and charged with argon three times. The above mixture was then stirred at 60 °C for 20 hours. After cooling to indoor temperature, the mixture solution was removed *via* rotary evaporation, and the residue was further purified *via* silica gel column chromatography using (PE: DCM = v/v 1/1) to afford white solid 131 mg with a yield of 75%. ^{1}H NMR (400 MHz, Chloroform-d) δ 9.24 (d, J = 6.2 Hz, 2H), 8.77 (s, 1H), 8.52 (d, J = 8.4 Hz, 1H), 8.03 (d, J = 1.9 Hz, 1H), 7.91 – 7.75 (m, 3H), 4.72 (d, J = 6.2 Hz, 4H), 3.22 (t, J = 8.0 Hz, 4H), 2.20 (dt, J = 13.3, 6.7 Hz, 2H), 1.85 (t, J = 7.8 Hz, 4H), 1.53 (h, J = 9.5, 7.4 Hz, 8H), 1.36 (t, J = 8.5 Hz, 8H), 1.18 – 0.91 (m, 36H), 0.85 (q, J = 6.6 Hz, 18H), 0.68 (p, J = 7.0 Hz, 12H). ^{13}C NMR (101 MHz, Chloroform-d) δ 187.19, 158.73, 158.07, 146.48, 146.41, 141.02, 140.92, 140.24, 137.21, 136.72, 136.17, 134.32, 133.30, 133.17, 128.37, 127.17, 125.71, 125.37, 123.49, 118.30, 114.26, 114.12, 113.88, 113.54, 112.56, 112.52, 67.71, 67.21, 54.76, 38.01, 30.90, 30.54, 30.28, 29.44, 29.34, 28.92, 28.69, 28.64, 28.60, 28.50, 28.38, 28.32, 27.01, 26.88, 24.48, 24.34, 21.78, 21.67, 21.45, 13.09, 12.99, 12.73. HRMS (MALDI-TOF): m/z: [M]+ calcd. for C92H106Br2N6O2S3Se2, 1742.4216; found,1744.8221. To a 25 mL round-bottom dry flask, compounds YBSe (87 mg, 0.05 mmol), SS (29 mg, 0.05 mmol), and $Pd(PPh_3)_4$ were dissolved with dry toluene (5 mL) under vacuum and flushed with dry nitrogen three times. Then, the reaction was carried out at 110 °C for 1 day. After that, the mixture was cooled, and water and chloroform were used for washing. After drying, the mixture was precipitated with methanol to receive the crude product after concentration. Afterwards, Soxhlet extraction was further performed to purify. With recrystallisation in acetonitrile, the production was achieved in a black solid (40 mg, 42%). 1H NMR (400 MHz, Chloroform-d) δ 9.34 – 8.34 (m, 3H), 8.23 – 7.68 (m, 3H), 7.67 – 7.27 (m, 3H), 7.14 – 6.38 (m, 3H), 4.81 (d, J = 41.8 Hz, 4H), 3.66 – 2.02 (m, 11H), 2.01 – 1.61 (m, 10H), 1.61 – 0.41 (m, 81H).

Photothermal performance

The photothermal performances of NPs and Mito-NPs in water with different concentrations (0.5 mL; from 0 to 50 μg mL^{-1}) were obtained by laser irradiation at 808 nm. The temperature changes under various treatments were recorded by an infrared thermal camera (FLUKE Ti480 PRO). According to the temperature curves, the photothermal conversion efficiencies (η) were calculated using the following equation:

$$\eta = \frac{hS\Delta T_{max} - Q_{Dis}}{I(1-10^{-A_{808}})}$$

where h is the heat transfer coefficient; S is the container area; ΔT_{max} is the maximum temperature variation, which refers from T_{max}-T_{surr} (T_{max} and T_{surr} are the maximum temperature and surroundings temperature); Q_{Dis} presents the heat related to the light absorbance by pure water; I is laser power density (1 W cm^{-2} here in this work); A_{808} presents the absorbance value of 808 nm in the tested sample; hS was calculated with the following equation:

$$\tau_S = \frac{m_D C_D}{hS}$$

where m_D presents the mass (0.5 g), C_D presents the heat capacity (4.2 J/g) of pure water used as the solvent, and τ_s was calculated with the following equation:

$$t = -\tau_S(\ln\theta)$$

where θ is the ratio of ΔT to ΔT_{max} during the natural cooling period.

Furthermore, the photothermal stability of Mito-NPs was determined through laser irradiation for 10 cycles (laser on/off), utilizing an indocyanine green solution with the same concentration to be a reference.

Fluorescence Quantum yield (QY) calculation

The QY of Mito-NPs were calculated by measuring the integrated fluorescence (900-1400 nm region) for five concentrations with various absorbance at 808 nm. Meanwhile, IR26 dye (QY = 0.5%) was chosen as a reference. Comparison of the slopes led to the determination of the QY of Mito-NPs. The QY was calculated in the following manner:

$$\mathrm{QY_{sample}} = \mathrm{QY_{ref}} \frac{\mathrm{slope_{sample}}}{\mathrm{slope_{ref}}} \left(\frac{\mathrm{n_{sample}}}{\mathrm{n_{ref}}}\right)^2$$

Where QY_{ref} is 0.5% and n_{sample} and n_{ref} present the refractive indices of water and dichloroethane, respectively.

Calculation details

The molecule was optimized at the b3lyp/6-31g(d) level using the Gaussian 16 program package *(69)*. The LUMO and HOMO were visualized using Multiwfn_3.8 and VMD_1.9.3. *(70)*. The density functional theory (DFT) was used to optimized the ground-state (S_0) geometry of the investigated molecule. Meanwhile, time-dependent DFT was further employed to optimize the excited-state geometry. The polarizable continuum model (PCM) was adopted to evaluate the

aqueous solvent environment, with the PBE0 functional and Def2-SVP basis set being selected for the calculations. All calculations were performed using Gaussian 16 software. Under aqueous conditions, the values of K_r, K_{nr}, and K_{isc} were obtained using the Molecular Materials Property Prediction Package (MOMAP) 2022B program *(71).*

Cell lines

Human osteosarcoma cells (143B), mouse osteosarcoma cells K7M2-wt (K7M2), and mouse bone marrow-derived dendritic cells (DC2.4) were provided by the Institute of Basic Medical Sciences, Chinese Academy of Medical Sciences (Beijing, China). Mouse fibroblast Cells (L929) were provided by Wuhan Union Hospital. 143B, K7M2, and L929 cells were also cultured in DMEM supplemented with 10% FBS, 1% penicillin and streptomycin in a humidified incubator containing 5% CO_2 at 37 °C.

***In vitro* cytotoxicity assay**

The cytotoxicity of Mito-NPs to cancer cells (143B and K7M2) and normal mouse fibroblast cells (L929) was evaluated by CCK8 assay. Briefly, cells were seeded in 96-well plates at a density of 4×10^3 cells per well and cultured in fresh culture medium at 37 °C overnight to achieve a 60-70% confluence. For the dark groups, the 143B or K7M2 cells were further incubated in fresh medium mixed with various concentrations of Mito-NPs (0, 3.12, 6.25, 12.5, 25, 50 μg mL^{-1}) for another 24 h incubation at 37 °C. The CCK-8 assay was used to determine relative cell viability. For the laser irradiation groups, when replacing with fresh medium mixed with various concentrations of Mito-NPs for another 4 h incubation at 37 °C, the cells were illuminated with 808 nm laser (0.33 W cm^{-2} and 1.0 W cm^{-2}, respectively) for 6 min. After another 24 h incubation, the CCK-8 assay was performed as above. Moreover, the dark cytotoxicity of Mito-NPs to L929 cells was also detected using the CCK-8 assay. Live/dead cell staining assay and apoptosis detection were performed by CLSM and FCM, respectively.

RNA sequencing and analysis

143B cells received different treatments, including PBS and Mito-NPs+0.33L, and then incubated for another 6 h. A Trizol reagent (Ambion, 260808) was used to extract the total RNA from the treated cells. High-throughput sequencing was performed by GENEWIZ of Life Sciences Co., Ltd. (3 sets of PBS and Mito-NPs+0.33L, respectively). A differential expression analysis was performed *via* the DESeq (v1.38.3) software. For differentially expressed genes (DEGs), the upregulated or downregulated genes were identified based on a P-value threshold of < 0.05.

Biosecurity

The major organs, mainly including the heart, liver, spleen, lung, and kidney, were collected from the treated mice for further H&E staining. Furthermore, the blood samples were collected at the end of treatments to test the main hematology markers and blood biochemical parameters. Blood and serum are collected for blood biochemistry tests and complete blood panel analysis by an automated spectrophotometric analyzer (n = 3).

Figs. S1 to S64

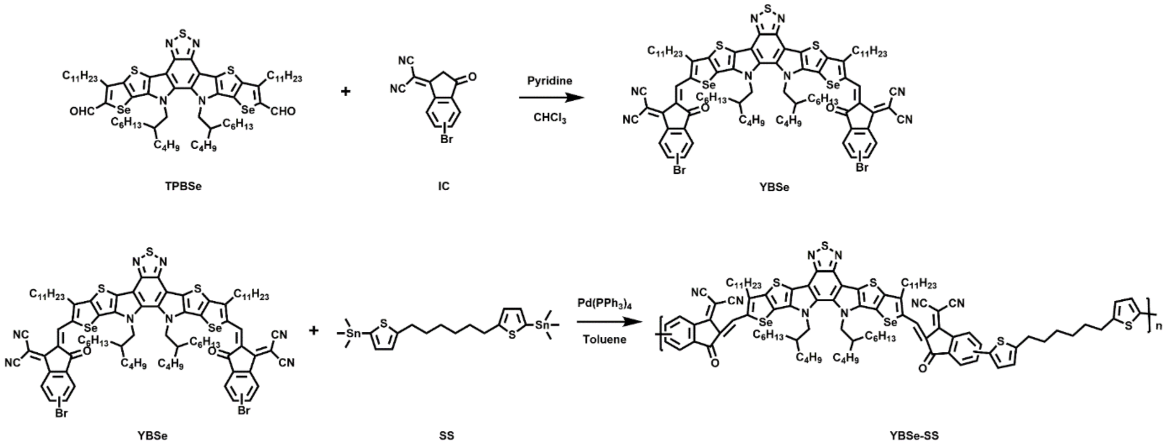

Fig. S1. Synthesis route of YBSe-SS.

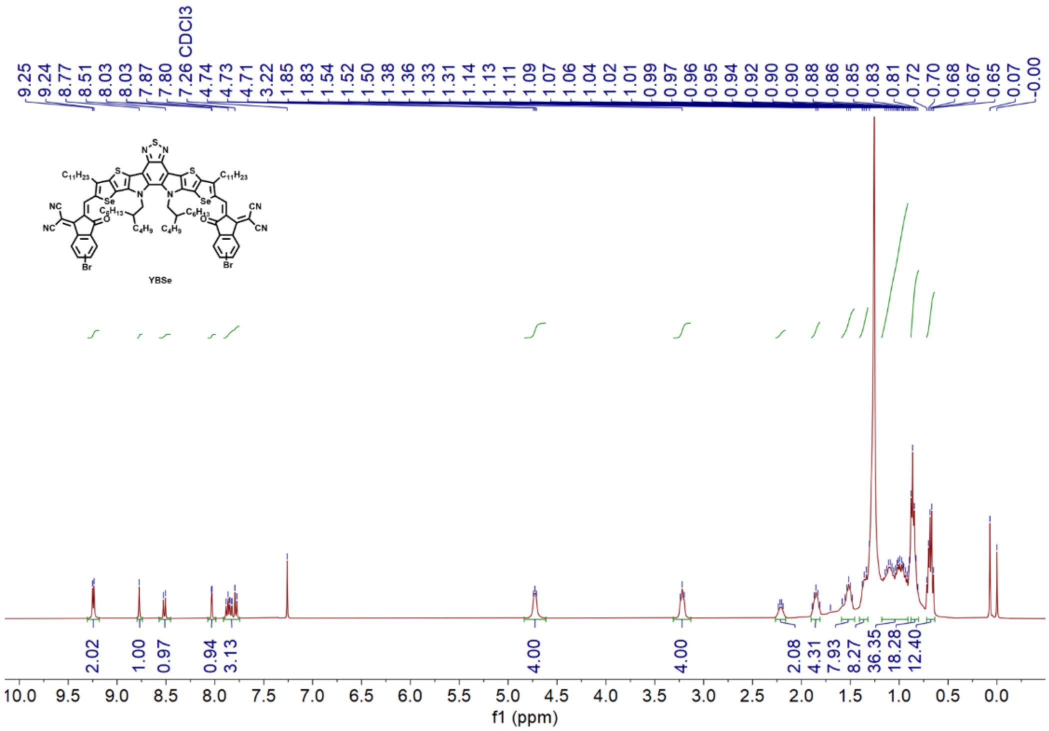

Fig. S2. ^{1}H NMR spectrum of YBSe in $CDCl_3$.

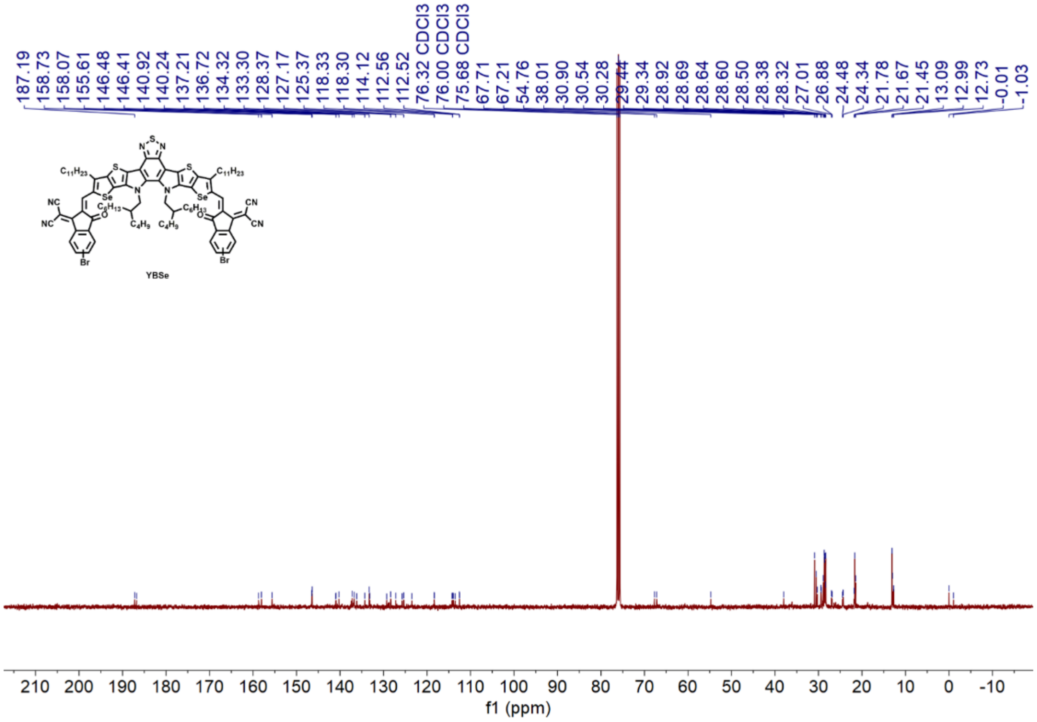

Fig. S3. ^{13}C NMR spectrum of YBSe in $CDCl_3$.

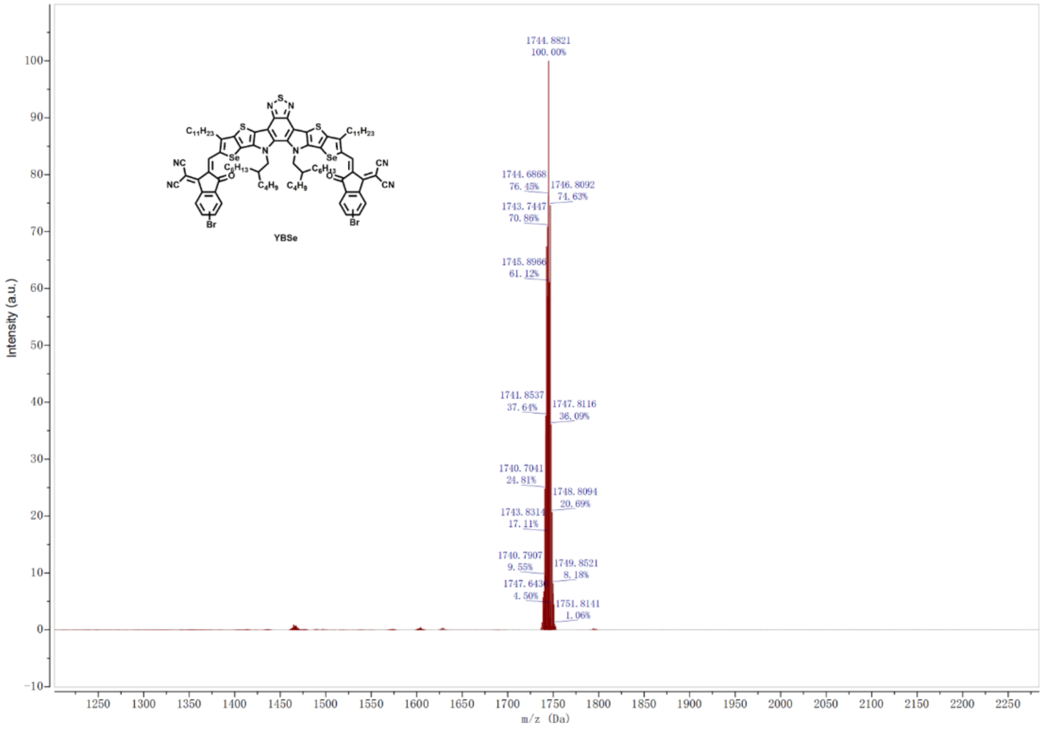

Fig. S4. HRMS spectrum of YBSe.

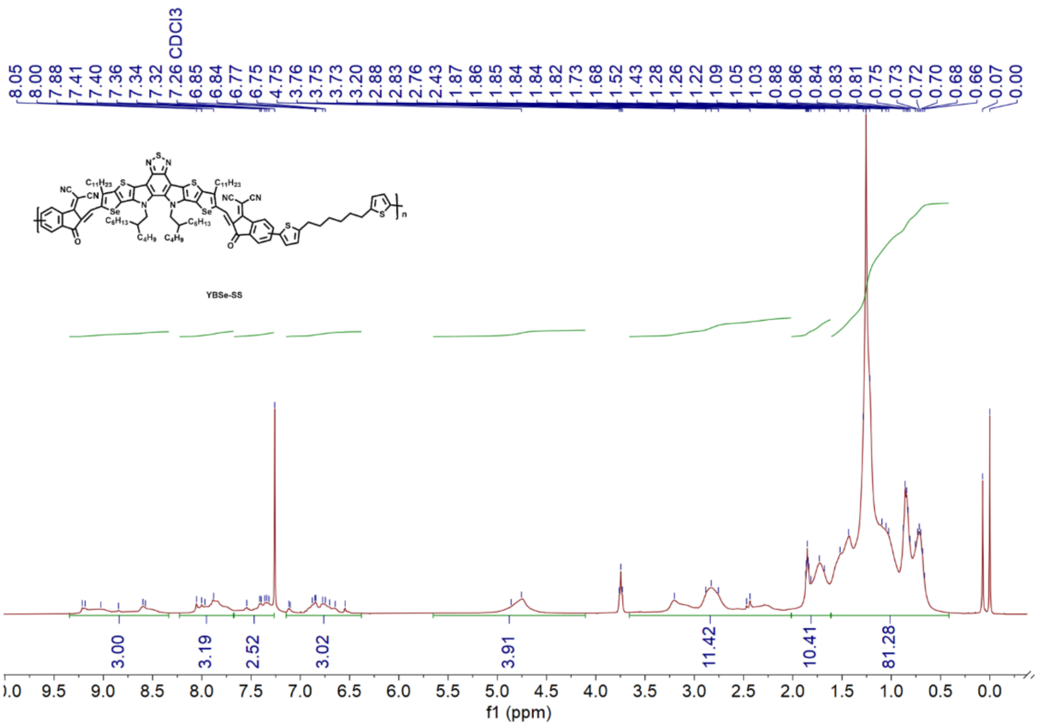

Fig. S5. ^{1}H NMR spectrum of YBSe-SS in $CDCl_3$.

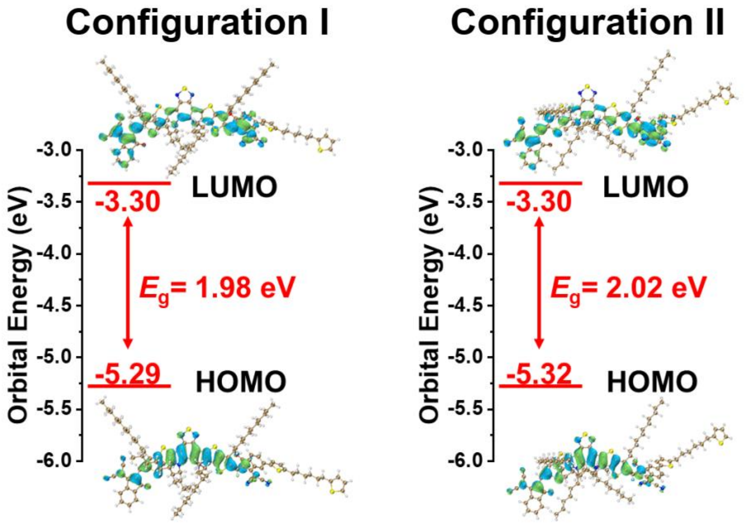

Fig. S6. HOMO and LUMO of YBSe-SS.

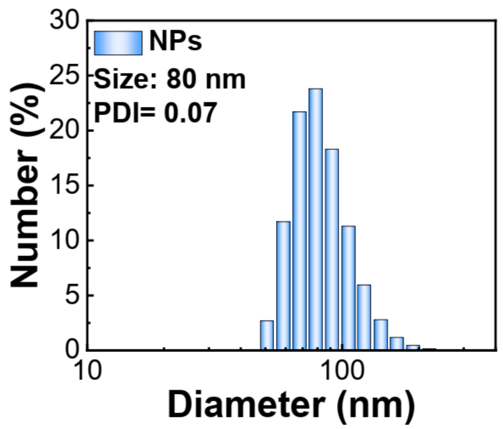

Fig. S7. Size of NPs.

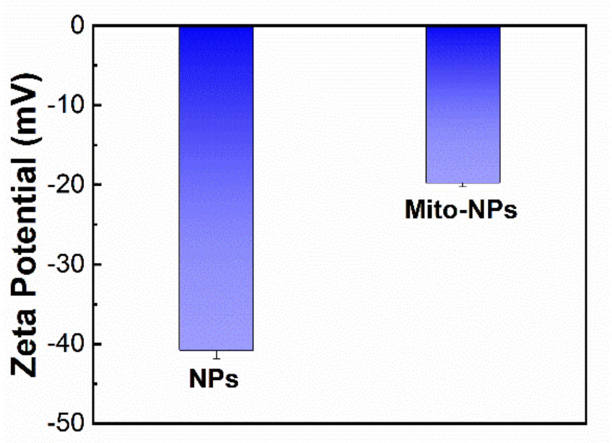

Fig. S8. Zeta potentials of NPs and Mito-NPs.

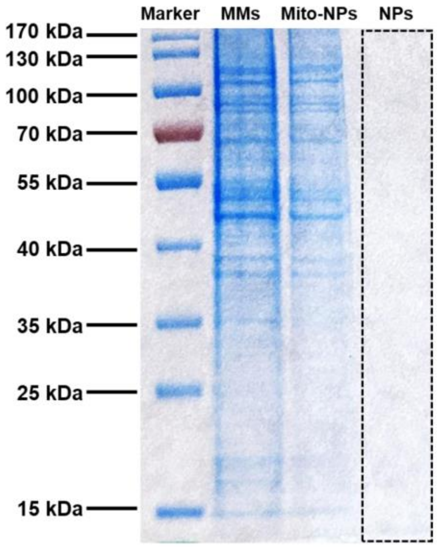

Fig. S9. SDS-PAGE protein analysis with Coomassie blue staining of mitochondrial membrane.

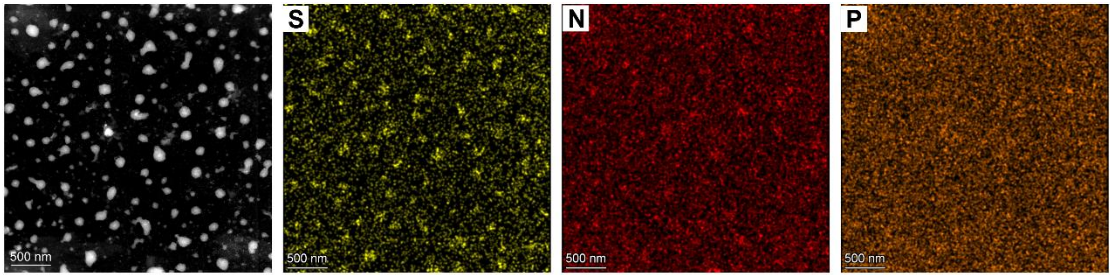

Fig. S10. TEM image (dark field) of Mito-NPs and the corresponding elemental mapping in the mitochondrial membrane.

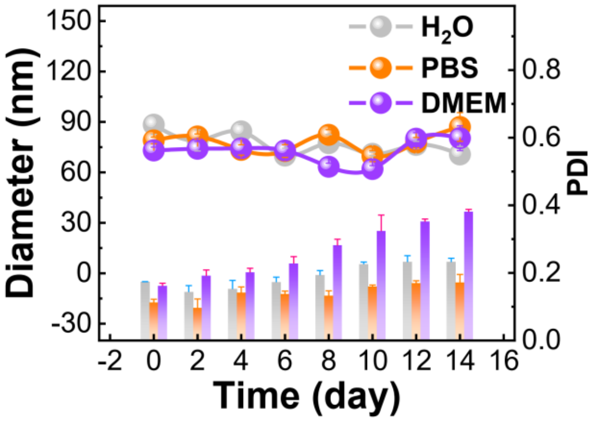

Fig. S11. Size distributions of Mito-NPs by DLS analysis over 14 days storage in various aqueous media.

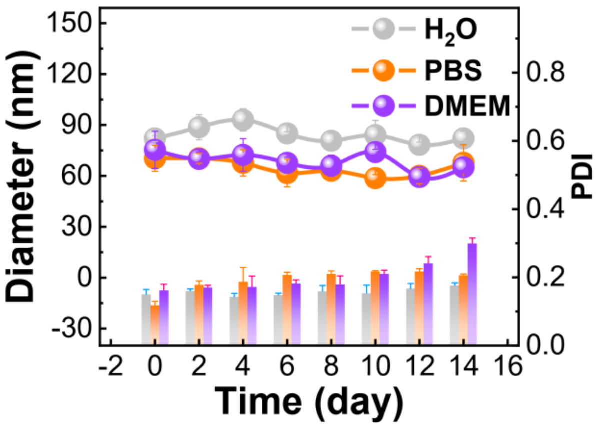

Fig. S12. Size distributions of NPs by DLS analysis over 14 days of storage in various aqueous media.

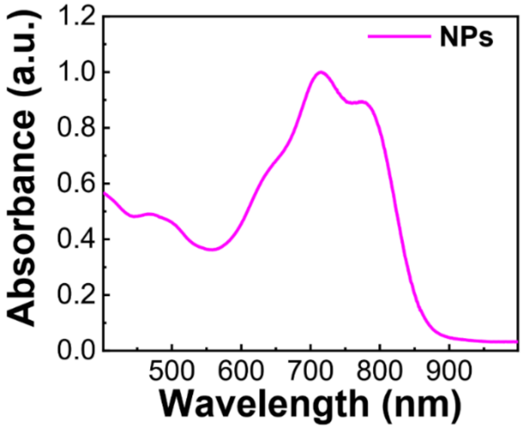

Fig. S13. Absorption spectra of NPs.

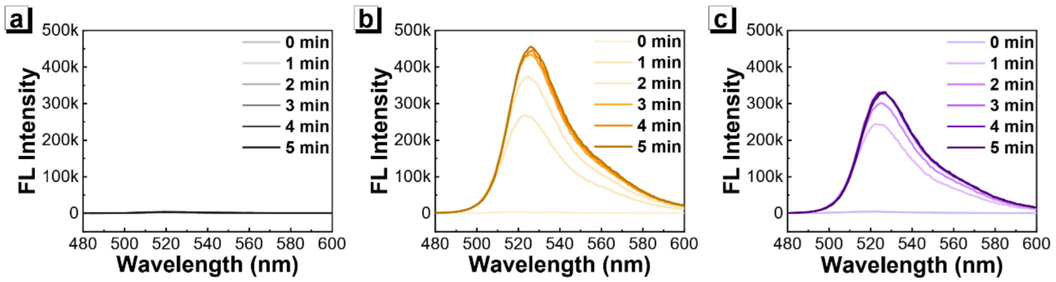

Fig. S14. Time-dependent fluorescence spectra of DCF for blank (a), NPs (b), and Mito-NPs (c) with 808 nm laser (0.33 W cm^{-2}) irradiation.

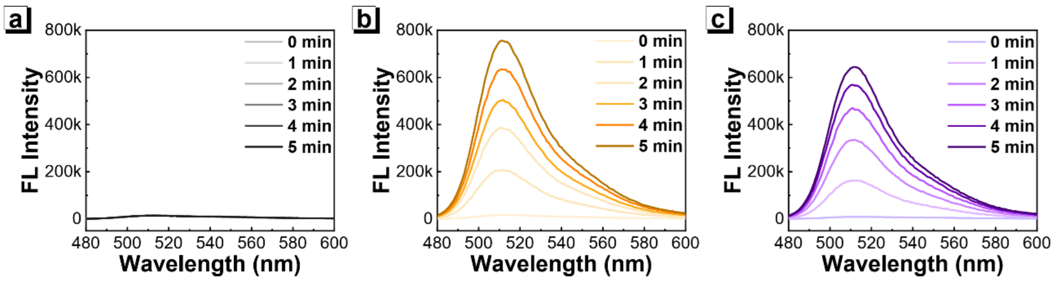

Fig. S15. Time-dependent fluorescence spectra of HPF for blank (a), NPs (b), and Mito-NPs (c) with 808 nm laser (0.33 W cm^{-2}) irradiation.

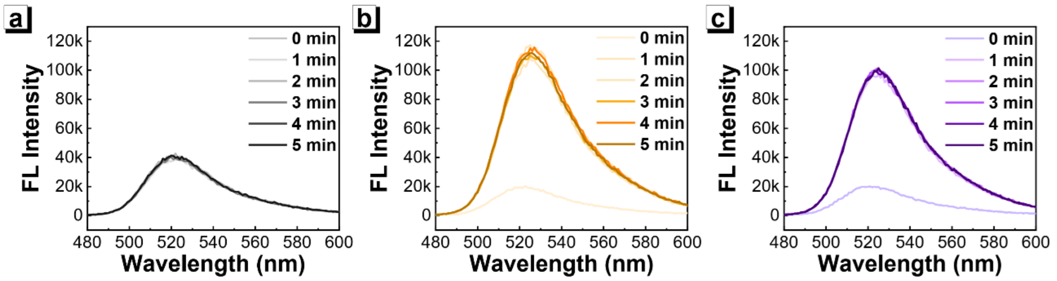

Fig. S16. Time-dependent fluorescence spectra of DHR123 for blank (a), NPs (b), and Mito-NPs (c) with 808 nm laser (0.33 W cm^{-2}) irradiation.

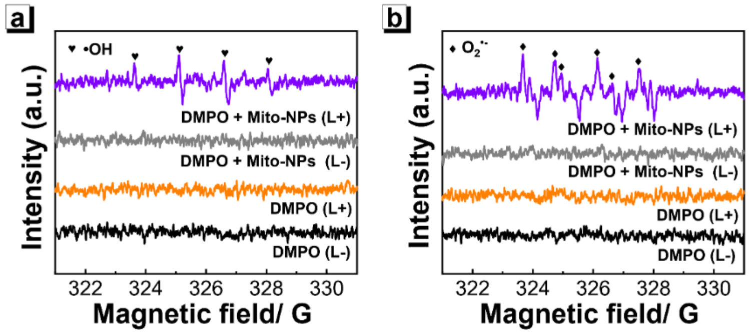

Fig. S17. ESR spectra of •OH (a) and O2$^{\bullet-}$ generation from Mito-NPs using DMPO probe under different conditions.

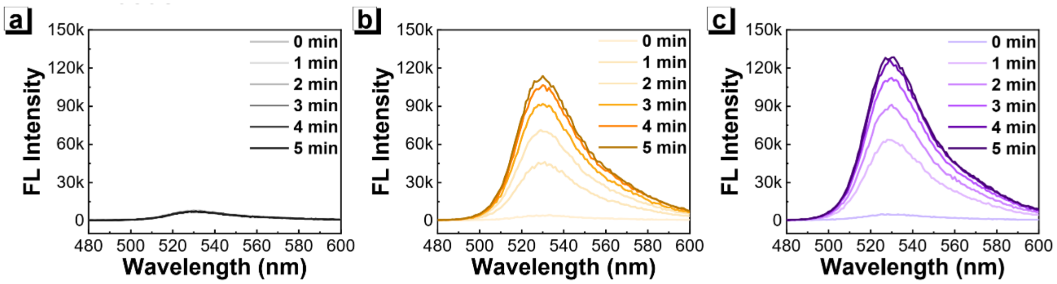

Fig. S18. Time-dependent fluorescence spectra of SOSG for blank (a), NPs (b), and Mito-NPs (c) with 808 nm laser (0.33 W cm^{-2}) irradiation.

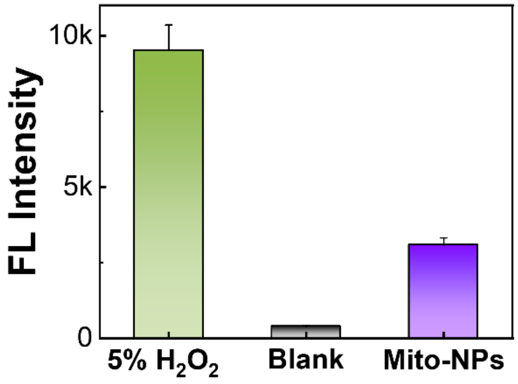

Fig. S19. Fluorescence intensity ROS Green™ H_2O_2 Probe in 5% H_2O_2 (as positive control), blank (PBS) and Mito-NPs with 808 nm laser (0.33 W cm^{-2}) irradiation for 5 min.

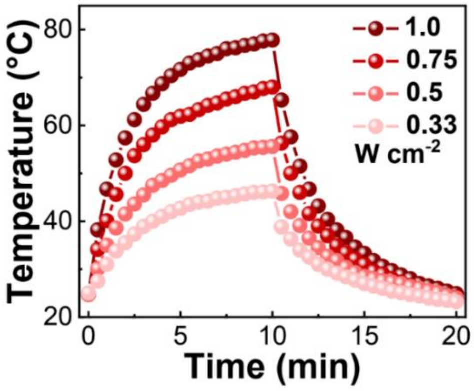

Fig. S20. Photothermal curves of Mito-NPs with various powers of 808 nm laser irradiation.

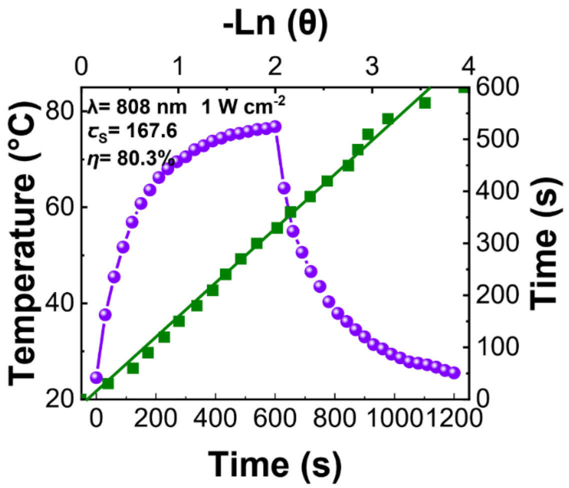

Fig. S21. Temperature profile of Mito-NPs under 808 nm laser (1 W cm^{-2}) illumination for 600 s and then cooling, and linear time data against –ln(θ) during the cooling period.

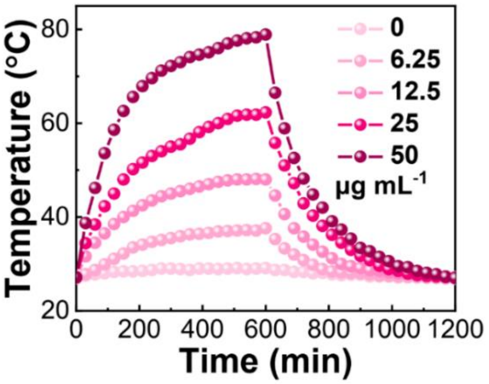

Fig. S22. Photothermal curves of NPs with various concentrations under 808 nm laser irradiation (1 W cm^{-2}).

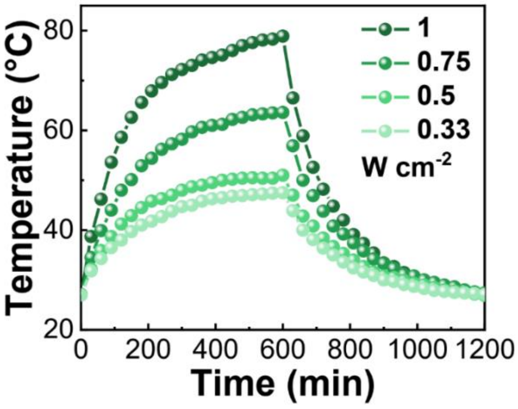

Fig. S23. Photothermal curves of NPs with various powers of 808 nm laser irradiation.

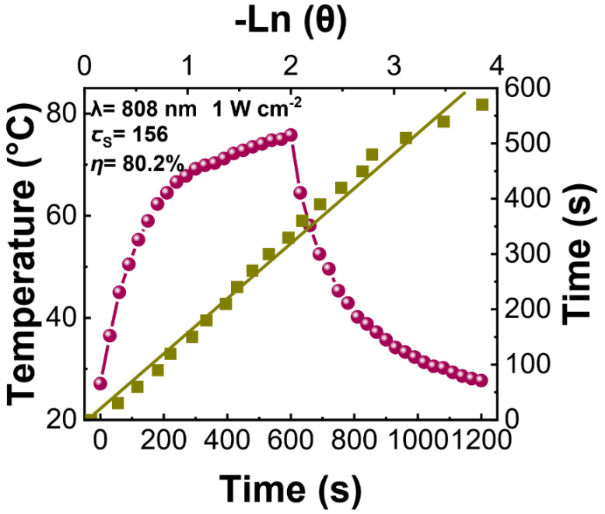

Fig. S24. Temperature profile of NPs under 808 nm laser (1 W cm^{-2}) illumination for 600 s and then cooling, and linear time data against –ln(θ) during the cooling period.

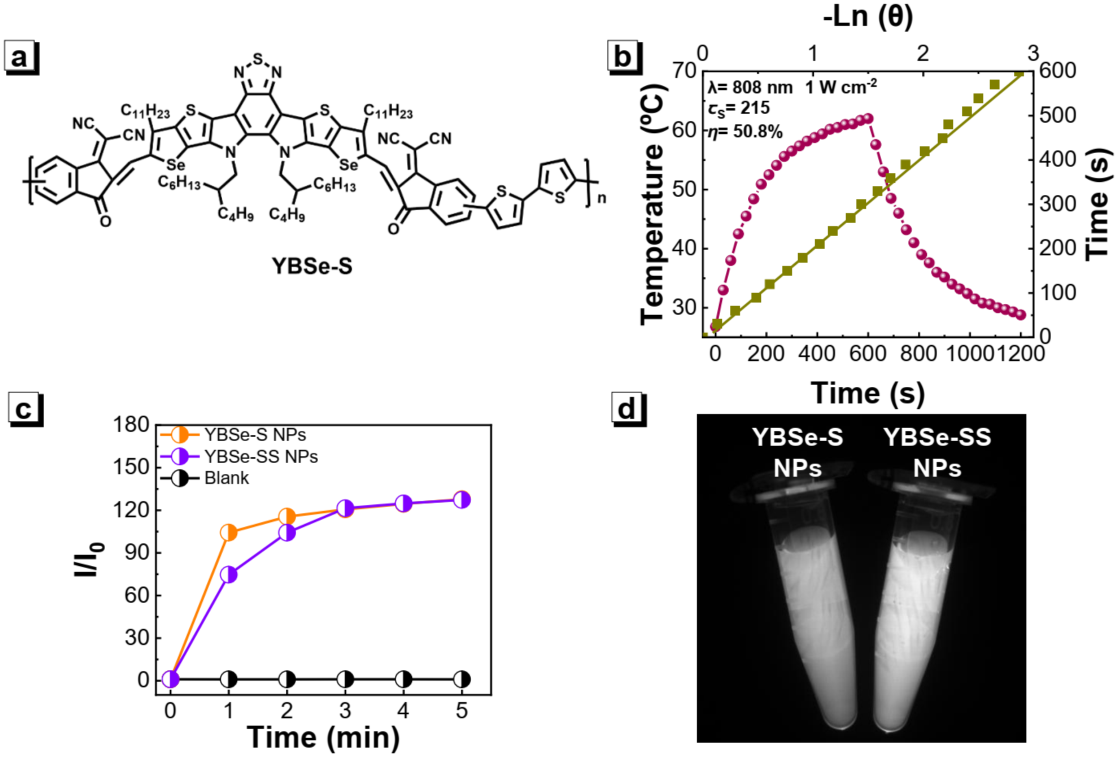

Fig. S25. (a) Molecular structure of YBSe-S. (b) PCE of YBSe-S NPs. Comparison of YBSe-S NPs and YBSe-SS NPs in ROS production (c) and fluorescence emission (d).

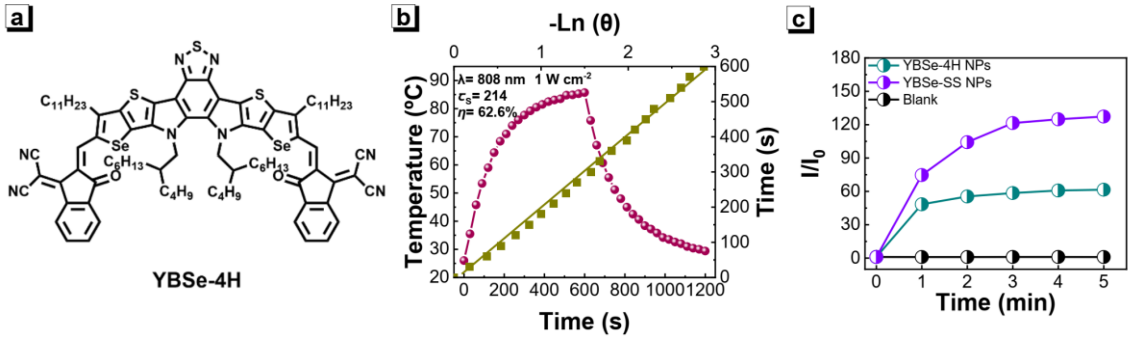

Fig. S26. (a) Molecular structure of YBSe-4H. (b) PCE of YBSe-4H NPs. (c) Comparison of YBSe-4H NPs and YBSe-SS NPs in ROS production.

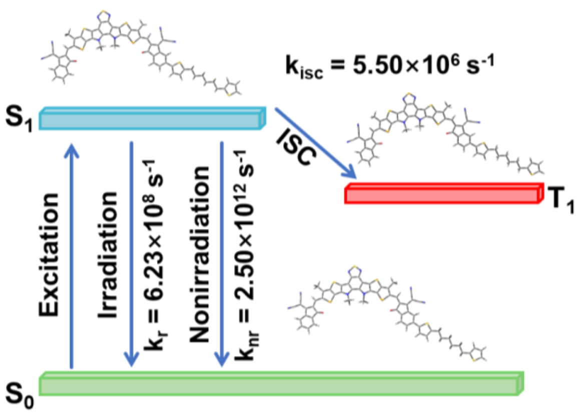

Fig. S27. Schematic diagram of the kinetic mechanism of YBSe-SS in aqueous solution.

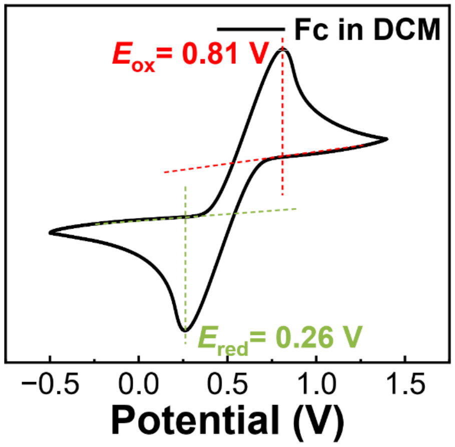

Fig. S28. Cyclic voltammograms of Fc/Fc^+ in DCM with 0.1 M $(n\text{-}Bu)_4N^+PF_6$ as a supporting electrolyte.

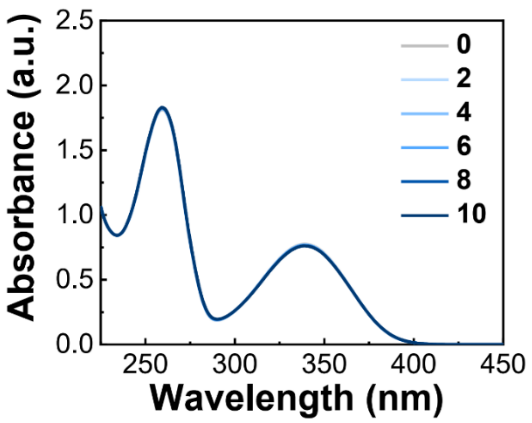

Fig. S29. Absorption spectra of NADH under 808 nm laser illumination for 10 min.

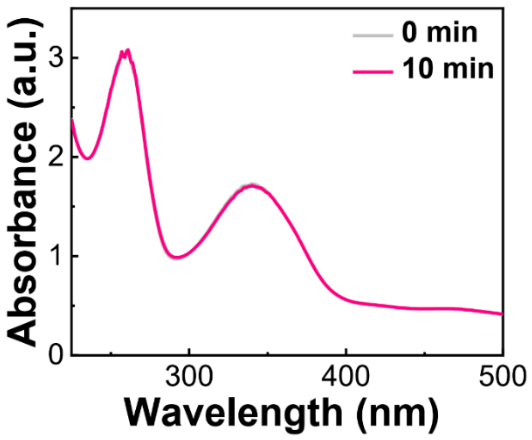

Fig. S30. Absorption spectra of NADH mixing with Mito-NPs in dark conditions for 0 and 10 min.

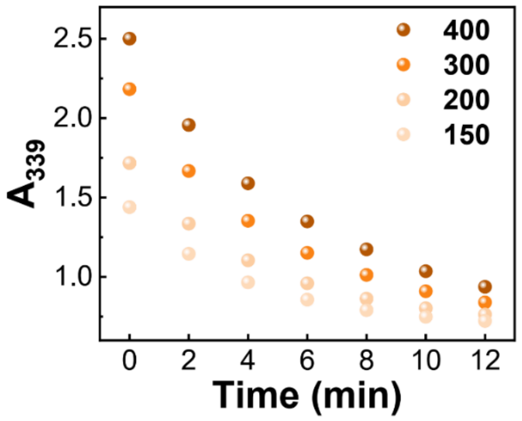

Fig. S31. Concentration dependence and time dependence of photocatalytic reaction of NADH with NPs (50 μg mL^{-1}) monitored by NADH absorption at 339 nm.

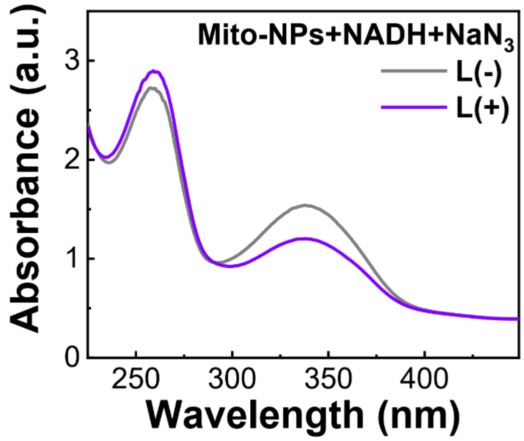

Fig. S32. Absorption spectra of NADH in the presence of Mito-NPs and 1 mM NaN_3.

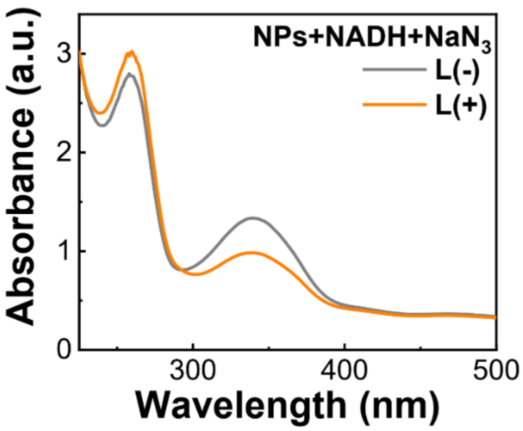

Fig. S33. Absorption spectra of NADH in the presence of NPs and 1 mM NaN_3.

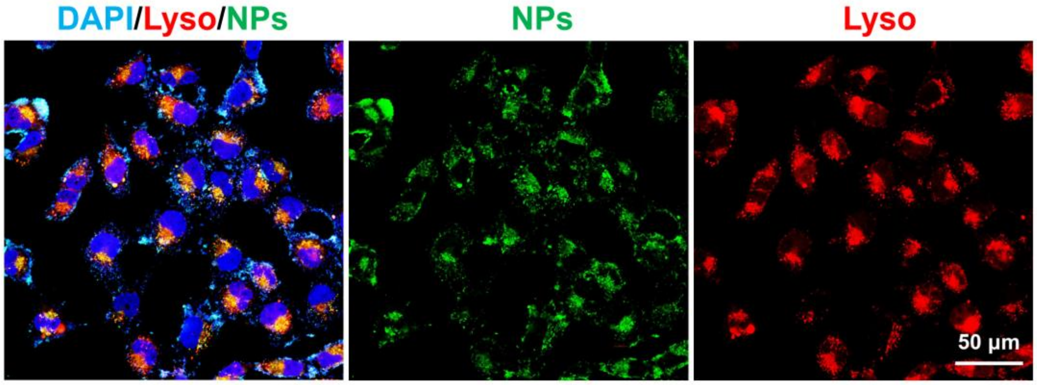

Fig. S34. Colocalization images of FITC-labelled NPs treated 143B cells with co-staining of Lyso-Tracker Red and DAPI.

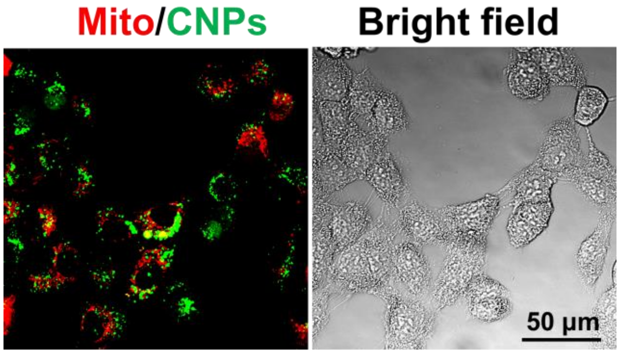

Fig. S35. Colocalization images of FITC-labelled 143B cell membrane-camouflaged NPs (CNPs) with co-staining of Mito-Tracker Red.

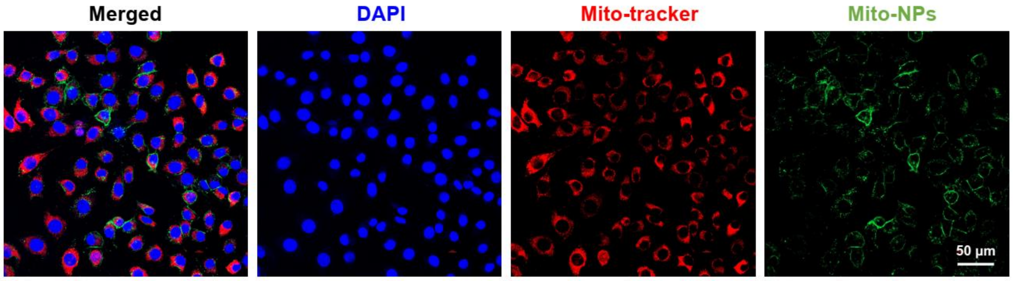

Fig. S36. Colocalization images of FITC-labelled Mito-NPs with co-staining of Mito-Tracker Red in L929 cells.

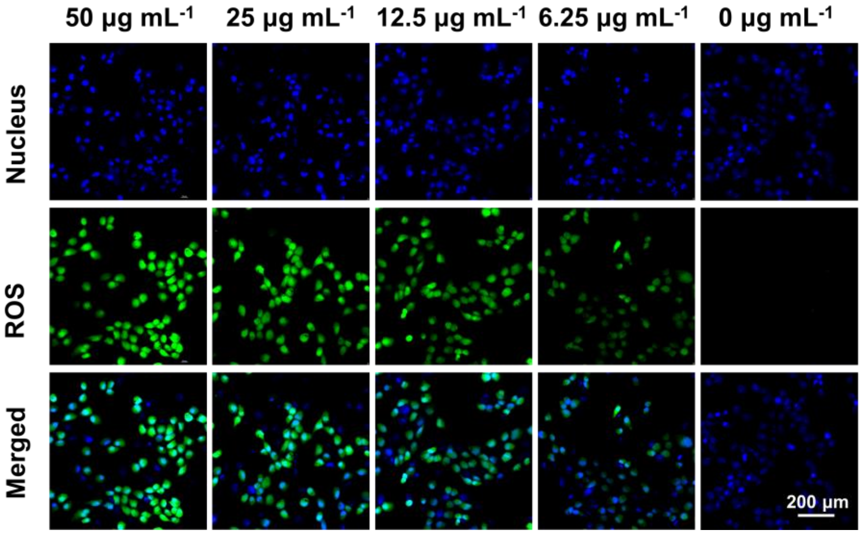

Fig. S37. Fluorescence imaging of ROS production in 143B cells treated with various concentrations of Mito-NPs. DCFH-DA is used as a ROS probe.

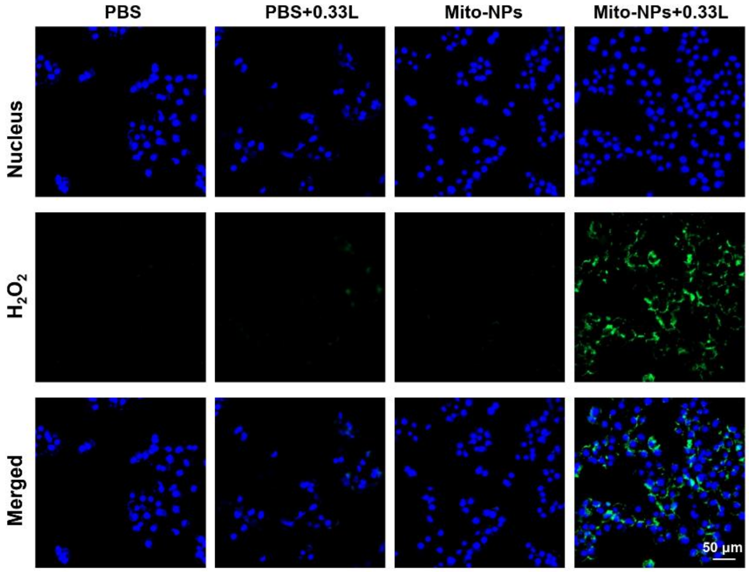

Fig. S38. Fluorescence imaging of H_2O_2 production in 143B cells treated with Mito-NPs and light irradiation. ROS Green^{TM} H_2O_2 Probe is used as a ROS probe.

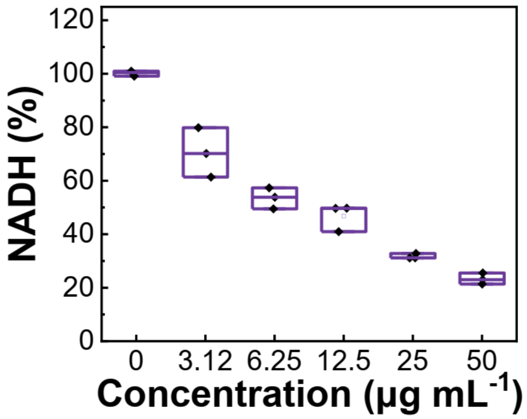

Fig. S39. Quantitative analysis of intracellular NADH contents with various concentrations of Mito-NPs under irradiation of 808 nm laser.

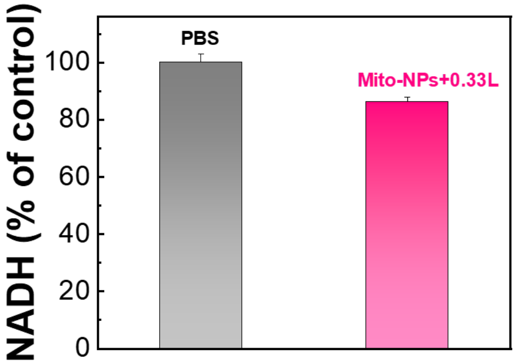

Fig. S40. Quantitative analysis of intracellular NADH contents with different treatments in L929 cells.

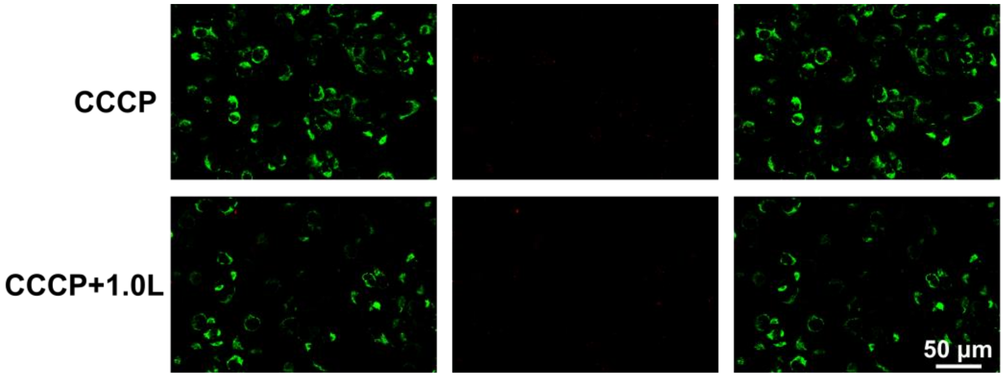

Fig. S41. JC-1 staining of 143B cells receiving carbonyl cyanide3 chlorophenylhydrazone (CCCP) as positive control.

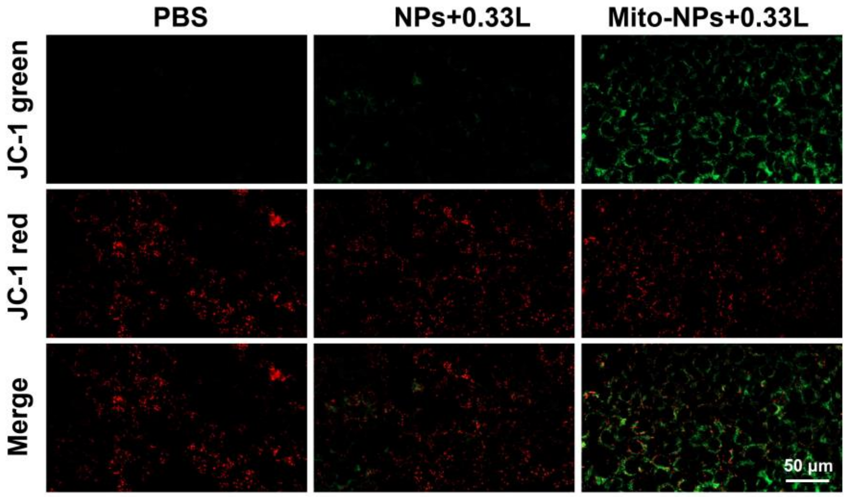

Fig. S42. JC-1 staining of 143B cells after various treatments.

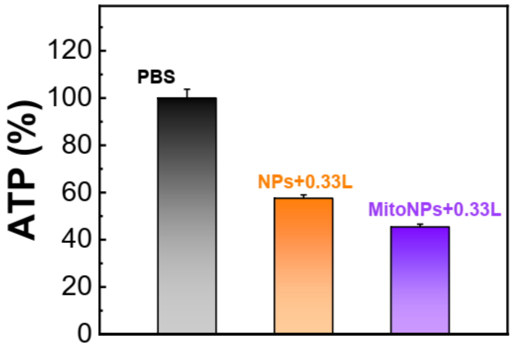

Fig. S43. Quantitative analysis of intracellular ATP levels in 143B cells with different treatments.

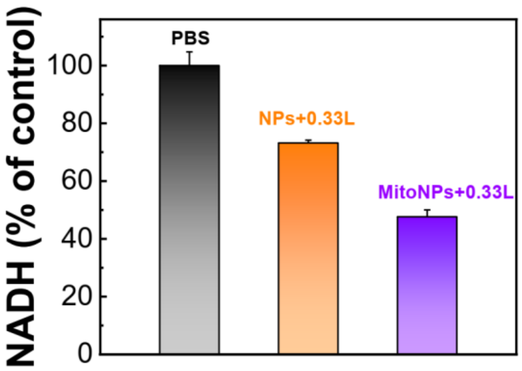

Fig. S44. Quantitative analysis of intracellular NADH levels in 143B cells with different treatments.

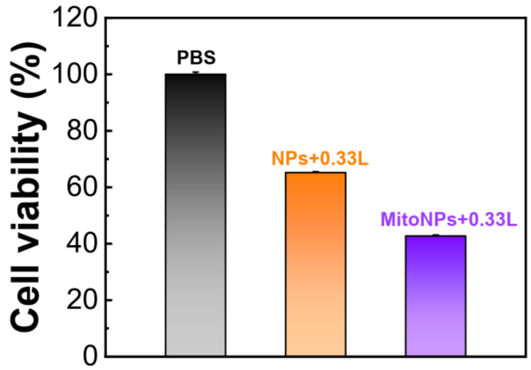

Fig. S45. Cell viability of 143B cells treated with various conditions.

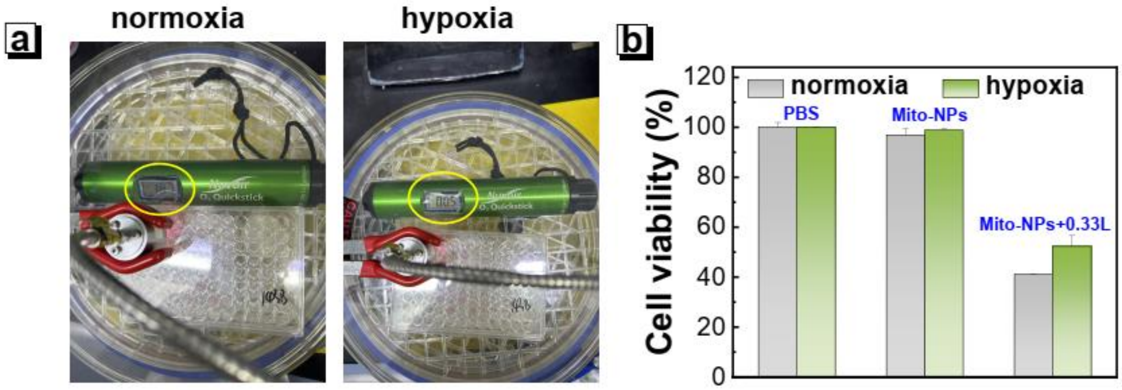

Fig. S46. (a) Device for normoxic and hypoxic environments. (b) Cell viability of 143B cells with various treatments in normoxic and hypoxic environments.

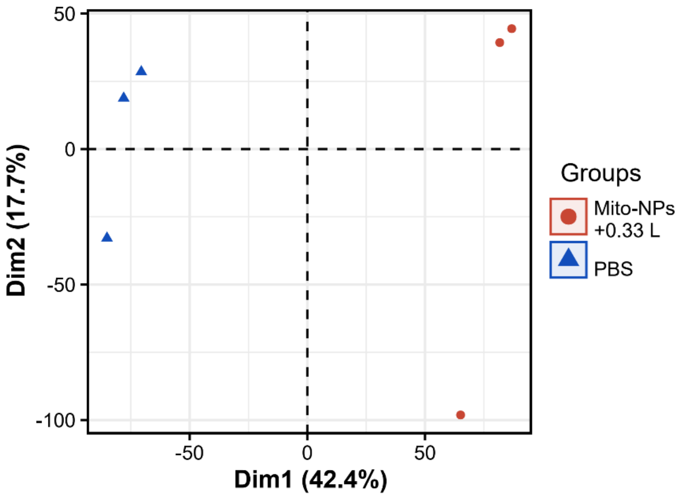

Fig. S47. PCA plot of clustered datasets between the two treatment groups.

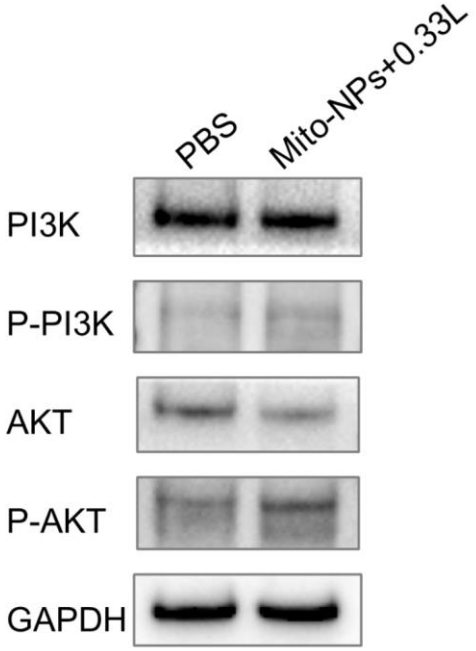

Fig. S48. PI3K-Akt protein expression by Western blot.

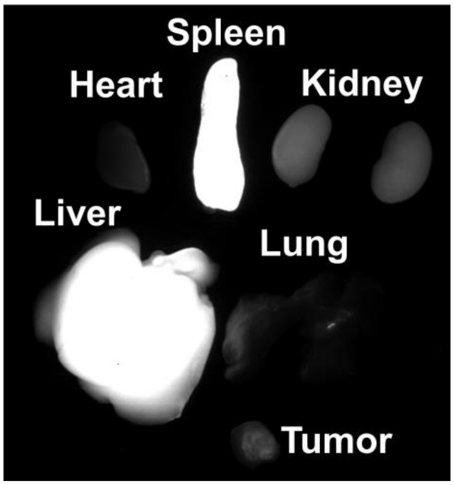

Fig. S49. *Ex vivo* NIR-II fluorescence images of different organs and tumor excised from 143B tumor-bearing mice at 48 h post-injection of Mito-NPs.

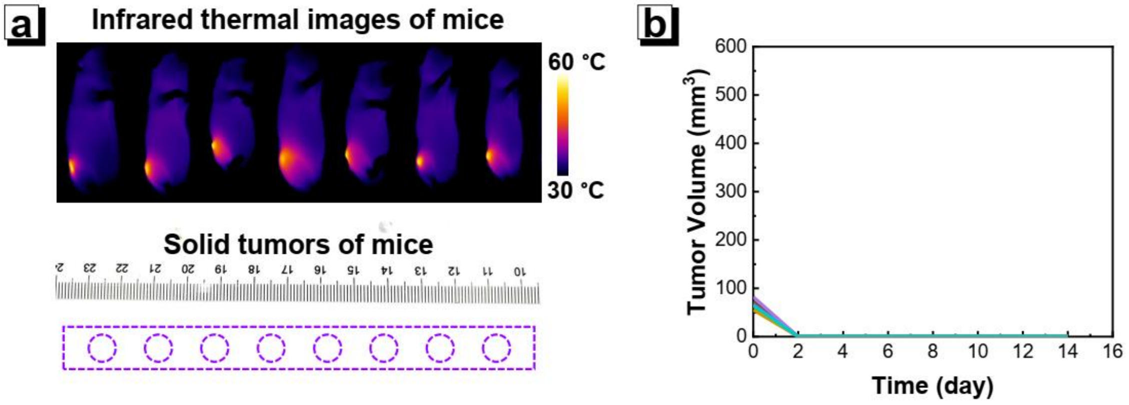

Fig. S50. *In vivo* phototherapy by Mito-NPs+1.0L (n=7). (a) Infrared thermal images of the mice with 808 nm laser irradiation during the first 5 min and digital images of tumors were separated from the treated mice at the end of the treatments. (b) Tumor growth curves of the treated mice within 14 days.

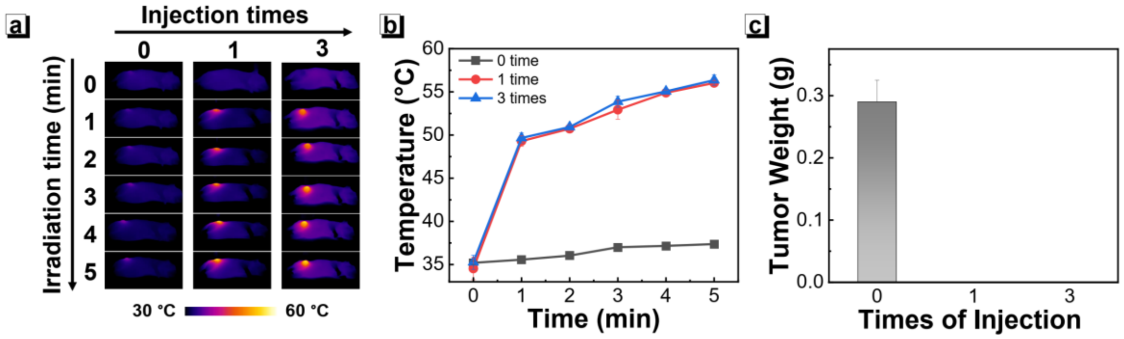

Fig. S51. Comparison of therapeutic effect with 1- or 3-times injection of Mito-NPs. (a and b) Infrared thermal images and temperatures of the mice with 808 nm laser irradiation during the first 5 min. (c) Tumor weight analysis of the treated mice after different treatments.

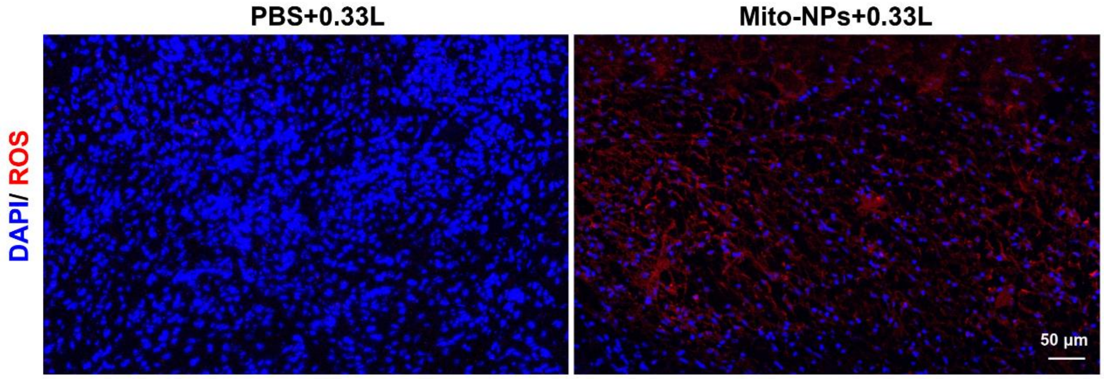

Fig. S52. The ROS detection in solid tumor via cryomicrotomy.

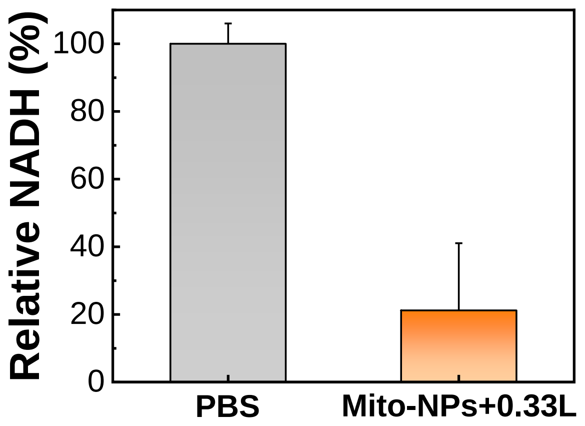

Fig. S53. Quantitative analysis of intracellular NADH contents in solid tumor cells after 808 nm laser irradiation (0.33 W cm^{-2}).

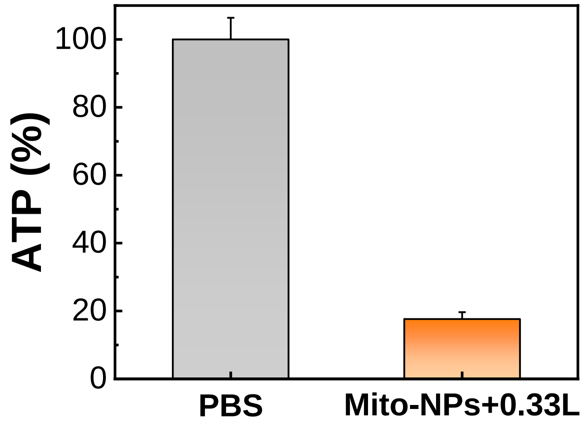

Fig. S54. Quantitative analysis of intracellular ATP contents in solid tumor cells after 808 nm laser irradiation (0.33 W cm^{-2}).

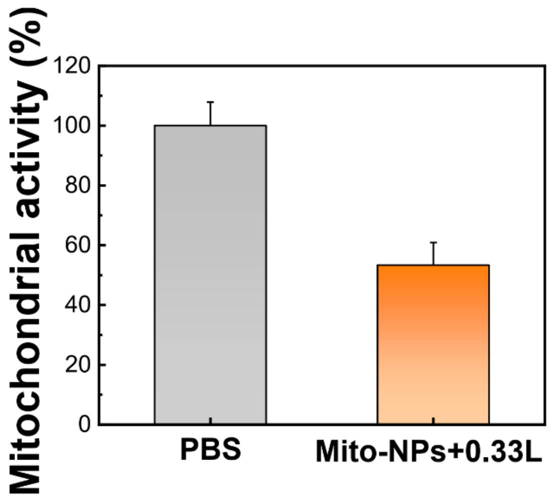

Fig. S55. Mitochondrial activity detection of cells in solid tumor after 808 nm laser irradiation (0.33 W cm^{-2}) using JC-1 staining.

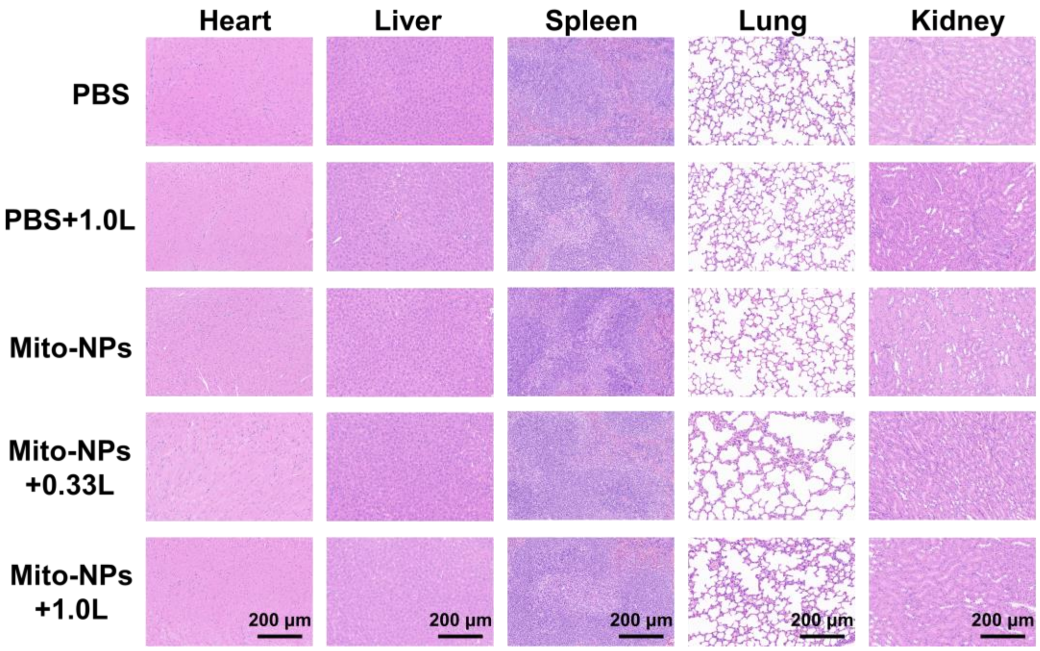

Fig. S56. H&E staining of the main organs from different groups after 14 days of treatment.

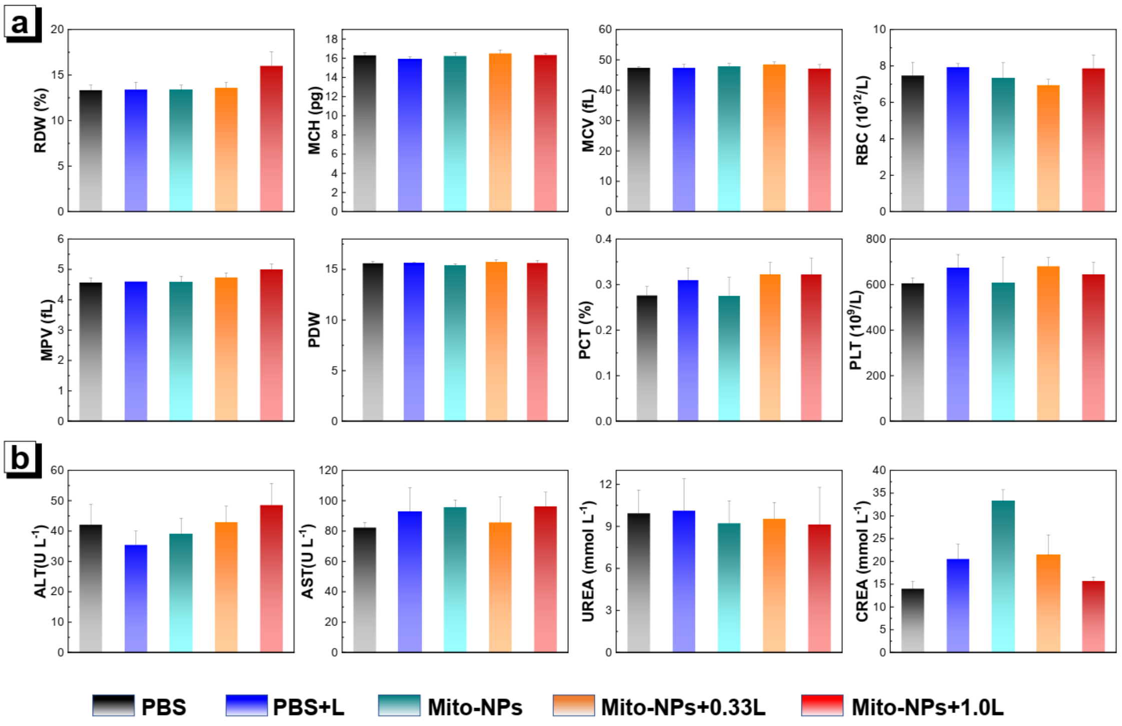

Fig. S57. Complete blood test (a) and biochemistry analysis (b) of the treated mice after 14 days of treatment.

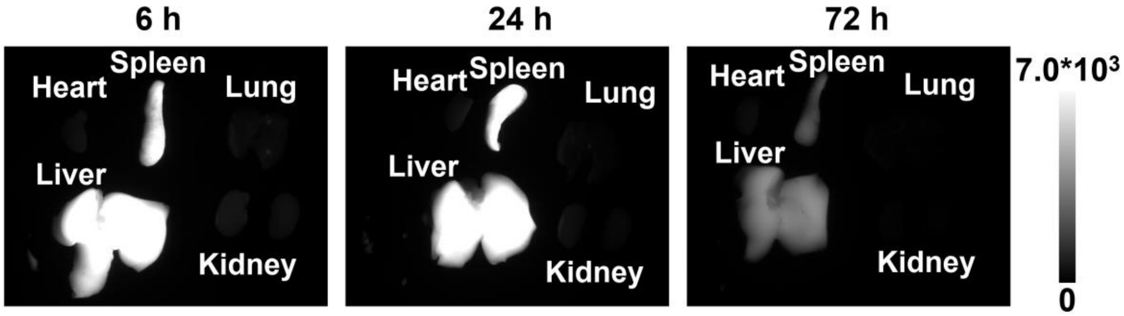

Fig. S58. *Ex vivo* NIR-II fluorescence images of the main organs excised from 143B tumor-bearing mouse at various post-injection times of Mito-NPs.

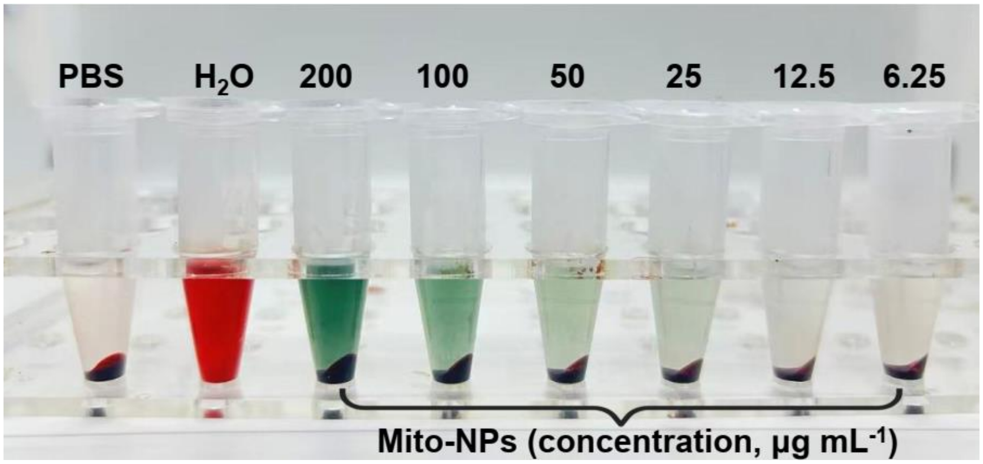

Fig. S59. Hemolytic test of Mito-NPs using PBS and water as controls.

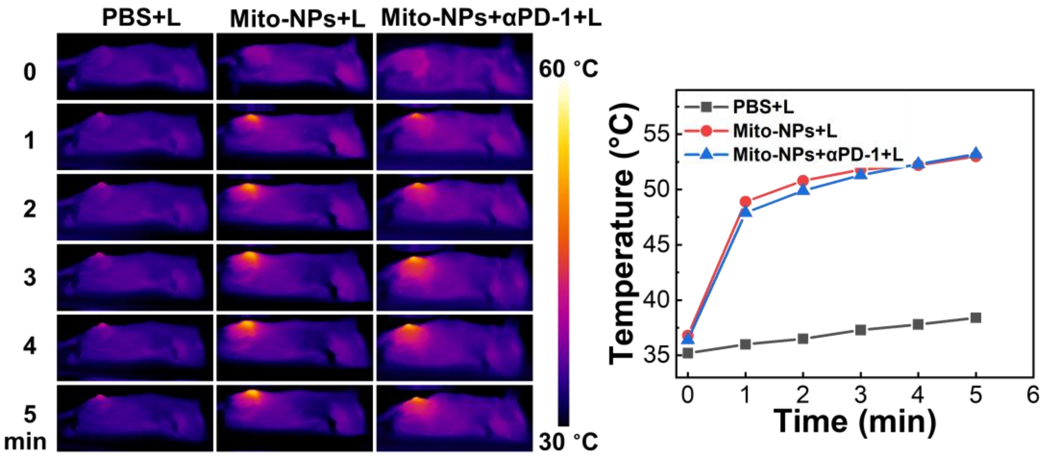

Fig. S60. Infrared thermal images of tumor sites of K7M2 tumor-bearing mice with 808 nm laser irradiation during the first 5 min and corresponding temperature profiles of tumors at 6 h post-injection of PBS or Mito-NPs.

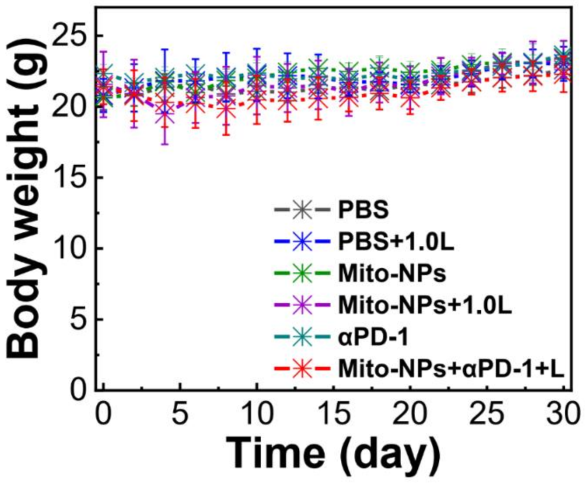

Fig. S61. Body weight changes of the treated mice.

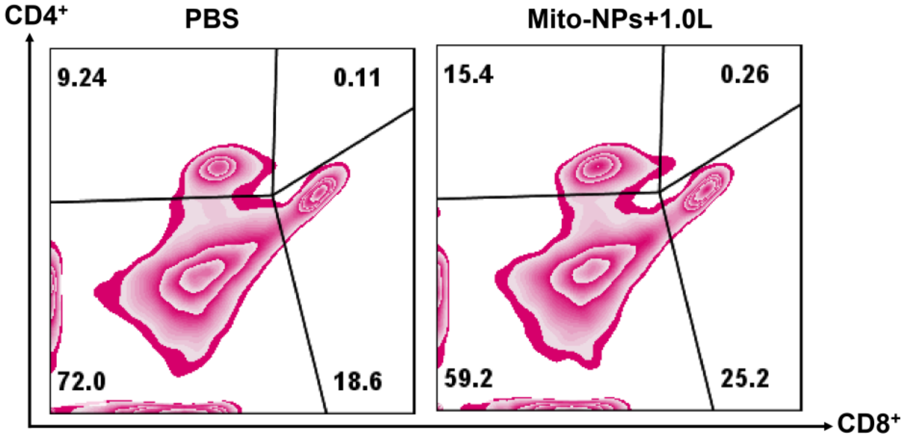

Fig. S62. Flow cytometry analysis of $CD8^+$ T cells ($CD3^+CD8^+$) in the spleen nodes of the treated mice after different treatments.

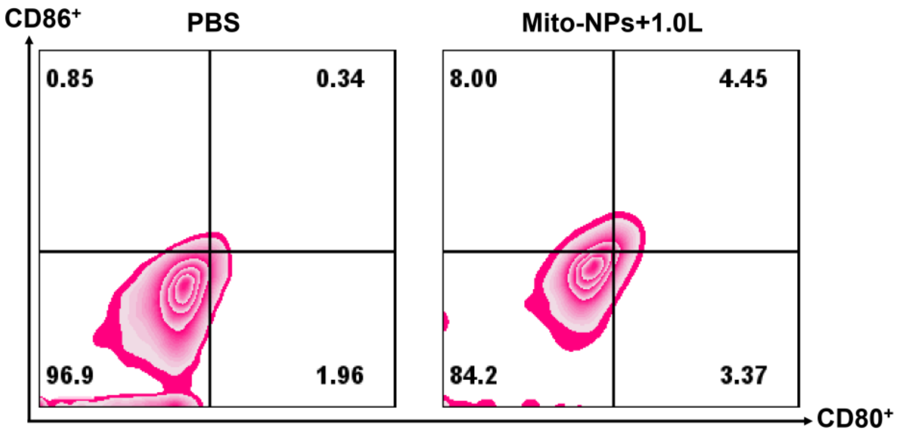

Fig. S63. Flow cytometry analysis of CD80$^+$/CD86$^+$ in the DC2.4 after different treatments.

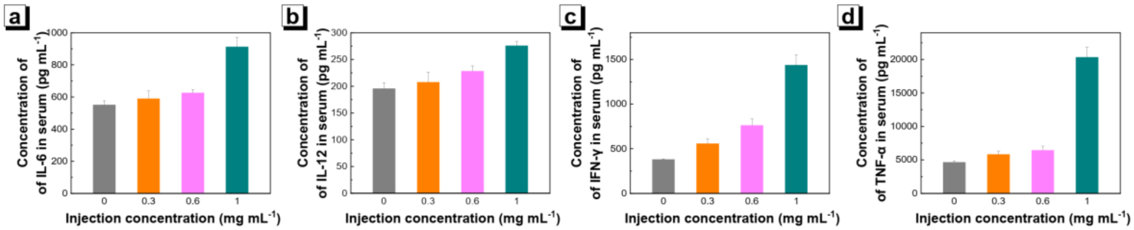

Fig. S64. ELISA assay of IL-6 (a), IL-12 (b), IFN-γ (c), and TNF-α (d) in the serum of the treated mice after different treatments.

Supplementary Tables

Table S1. Summary of recently reported NIR-II emissive materials.

**Agents**[a]		**$\lambda_{abs}/\lambda_{em}$ (nm)**	**QY (%)**[b]	**References**
Organic small molecule	TPC	773/921	9.8	*(24)*
	CTB1125	895/1125	4.84	*(75)*
	AS1	730/935	4.7	*(76)*
	4TT-PBPT	700/1026	1.94	*(77)*
	2TT-BBTD	773/1004	0.19	*(78)*
Conjugated polymer	IR-FTGP	784/1068	1.4	*(79)*
	IR-FTAP	733/1048	5.3	*(79)*
	IDS-IC	715/934	1.7	*(80)*
	m-PBTQ2F	931/1103	2.2	*(81)*
	m-PBTQ4F	946/1123	3.2	*(81)*
	PQTz-TT	782/1060	2.6	*(82)*
	PQTz-fTT	754/1062	2.7	*(82)*
	AIE Pdots	808/1319	1.7	*(83)*
	BTFQ	960/1113	0.63	*(84)*
	BETT-2	915/1240	0.13	*(85)*
	YBSe-SS	775/916	2.7	*This work*

a: NPs in H_2O; b: calculated to IR26.

Table S2. The rate constants of YBSe-SS and YBSe-S.

	K_{nr} ($\times 10^{12}$ s^{-1})	K_r ($\times 10^{8}$ s^{-1})	K_{isc} ($\times 10^{6}$ s^{-1})
YBSe-SS	2.50	6.23	5.50
YBSe-S	1.01	2.35	-

Table S3. The pathology score based on H&E of tumor tissues was calculated according to the four-level classification system in International Harmonization of Nomenclature and Diagnostic Criteria for Lesions in Rats and Mice (INHAND).

Groups	Inflammation	Bleeding index	Hematoma Index	Necrosis
PBS	1	1	1	1
PBS+L	1	1	1	1
Mito-NPs	1	1	1	1
Mito-NPs+0.33L	2	1	1	2
Mito-NPs+1.0L	0	0	0	4

REFERENCES AND NOTES

1. Z. Zhang, Y. Du, X. Shi, K. Wang, Q. Qu, Q. Liang, X. Ma, K. He, C. Chi, J. Tang, B. Liu, J. Ji, J. Wang, J. Dong, Z. Hu, J. Tian, NIR-II light in clinical oncology: Opportunities and challenges. *Nat. Rev. Clin. Oncol.* **21**, 449–467 (2024).

2. M. He, M. Xiao, R. Wang, J. Fan, X. Peng, W. Sun, Phototherapeutic nanoagents for cancer immunotherapy. *Prog. Mater. Sci.* **147**, 101347 (2025).

3. Q. Shen, G. Song, H. Lin, H. Bai, Y. Huang, F. Lv, S. Wang, Sensing, imaging, and therapeutic strategies endowing by conjugate polymers for precision medicine. *Adv. Mater.* **36**, e2310032 (2024).

4. Y. Wang, K. Ma, M. Kang, D. Yan, N. Niu, S. Yan, P. Sun, L. Zhang, L. Sun, D. Wang, H. Tan, B. Z. Tang, A new era of cancer phototherapy: Mechanisms and applications. *Chem. Soc. Rev.* **53**, 12014–12042 (2024).

5. P.-Z. Liang, L.-L. Ren, Y.-H. Yan, Z. Li, F.-Y. Yang, T.-B. Ren, L. Yuan, X.-B. Zhang, Activatable photosensitizer prodrug for self-amplified immune therapy via pyroptosis. *Angew. Chem. Int. Ed.* **64**, e202419376 (2025).

6. S. Zhen, Z. Xu, M. Suo, T. Zhang, M. Lyu, T. Li, T. Zhang, M. Li, Z. Zhao, B. Z. Tang, NIR-II AIE liposomes for boosting type-I photodynamic and mild-temperature photothermal therapy in breast cancer treatment. *Adv. Mater.* **37**, e2411133 (2024).

7. J. Kim, S. Lee, Y. Kim, M. Choi, I. Lee, E. Kim, C. G. Yoon, K. Pu, H. Kang, J. S. Kim, In situ self-assembly for cancer therapy and imaging. *Nat. Rev. Mater.* **8**, 710–725 (2023).

8. Y. Wan, W. Chen, Y. Liu, K. W. Lee, Y. Gao, D. Zhang, Y. Li, Z. Huang, J. Luo, C.-S. Lee, S. Li, Neutral cyanine: Ultra-stable NIR-II merocyanines for highly efficient bioimaging and tumor-targeted phototheranostics. *Adv. Mater.* **36**, e2405966 (2024).

9. J. An, S. Tang, G. Hong, W. Chen, M. Chen, J. Song, Z. Li, X. Peng, F. Song, W. H. Zheng, An unexpected strategy to alleviate hypoxia limitation of photodynamic therapy by biotinylation of photosensitizers. *Nat. Commun.* **13**, 2225 (2022).

10. M. Liu, Y. Chen, Y. Guo, H. Yuan, T. Cui, S. Yao, S. Jin, H. Fan, C. Wang, R. Xie, W. He, Z. Guo, Golgi apparatus-targeted aggregation-induced emission luminogens for effective cancer photodynamic therapy. *Nat. Commun.* **13**, 2179 (2022).

11. J. Sun, X. Cai, C. Wang, K. Du, W. Chen, F. Feng, S. Wang, Cascade reactions by nitric oxide and hydrogen radical for anti-hypoxia photodynamic therapy using an activatable photosensitizer. *J. Am. Chem. Soc.* **143**, 868–878 (2021).

12. J. Huang, C. Zhang, X. Wang, X. Wei, K. Pu, Near-infrared photodynamic chemiluminescent probes for cancer therapy and metastasis detection. *Angew. Chem. Int. Ed.* **62**, e202303982 (2023).

13. Y. Tang, Y. Li, C. He, Z. Wang, W. Huang, Q. Fan, B. Liu, NIR-II-excited off-on-off fluorescent nanoprobes for sensitive molecular imaging in vivo. *Nat. Commun.* **16**, 278 (2025).

14. L. Kan, Y. Zhang, Y. Luo, Y. Wei, J. Zhong, Y. Gao, Y. Liu, K. Wang, S. Li, Near-infrared emissive π-conjugated oligomer nanoparticles for three- and four-photon deep-brain microscopic imaging beyond 1700 nm excitation. *ACS Nano* **18**, 26828–26838 (2024).

15. M. Ma, Y. Zhang, K. Pu, W. Tang, Nanomaterial-enabled metabolic reprogramming strategies for boosting antitumor immunity. *Chem. Soc. Rev.* **54**, 653–714 (2025).

16. S. Zeng, J. Wang, H. Kang, H. Li, X. Peng, J. Yoon, Photon-driven dye induction pyroptosis: An emerging anti-tumor immunotherapy paradigm. *Angew. Chem. Int. Ed.* **64**, e202417899 (2024).

17. Y. Gao, Y. Liu, X. Li, H. Wang, Y. Yang, Y. Luo, Y. Wan, C. S. Lee, S. Li, X. H. Zhang, A stable open-shell conjugated diradical polymer with ultra-high photothermal conversion efficiency for NIR-II photo-immunotherapy of metastatic tumor. *Nanomicro Lett.* **16**, 21 (2023).

18. Y. Wang, J. Zhang, Q. Xu, W. Tu, L. Wang, Y. Xie, J. Z. Sun, F. Huang, H. Zhang, B. Z. Tang, Narrowband clusteroluminescence with 100% quantum yield enabled by through-space conjugation of asymmetric conformation. *Nat. Commun.* **15**, 6426 (2024).

19. W. Zhang, M. Kang, X. Li, Y. Pan, Z. Li, Y. Zhang, C. Liao, G. Xu, Z. Zhang, B. Z. Tang, Z. Xu, D. Wang, Fiber Optic-mediated type I photodynamic therapy of brain glioblastoma based on an aggregation-induced emission photosensitizer. *Adv. Mater.* **36**, e2410142 (2024).

20. L. Q. Xu, J. Zhang, J. Z. Sun, H. Zhang, B. Z. Tang, Efficient organic emitters enabled by ultrastrong through-space conjugation. *Nat. Photonics* **18**, 1185–1194 (2024).

21. C. Li, M. Yao, G. Jiang, L. Feng, Y. Wu, R. Sha, Y. Li, B. Z. Tang, J. Wang, Side chain phenyl isomerization-induced spatial conjugation for achieving efficient near-infrared II phototheranostic agents. *Angew. Chem. Int. Ed.* **64**, e202419785 (2025).

22. J. Huang, J. Liu, J. Wu, M. Xu, Y. Lin, K. Pu, Near-infrared chemiluminophore switches photodynamic processes via protein complexation for biomarker-activatable cancer therapy. *Angew. Chem. Int. Ed.* **64**, e202421962 (2025).

23. J. Yuan, H. Yang, W. Huang, S. Liu, H. Zhang, X. Zhang, X. Peng, Design strategies and applications of cyanine dyes in phototherapy. *Chem. Soc. Rev.* **54**, 341–366 (2025).

24. Y. Wang, J. Zhang, Y. Wang, J. Yu, Y. Gao, Y. Yang, X. Li, H. Wang, S. Li, A NIR-II-emissive organic nanomedicine with biomimetic engineering for high-contrast targeted bioimaging and multiple phototherapies of pancreatic tumors. *Adv. Funct. Mater.* **34**, 2406483 (2024).

25. M. Li, Y. Xu, X. Peng, J. S. Kim, From low to no O_2-dependent hypoxia photodynamic therapy (hPDT): A new perspective. *Acc. Chem. Res.* **55**, 3253–3264 (2022).

26. H. Liu, Z. Li, X. Zhang, Y. Xu, G. Tang, Z. Wang, Y. Y. Zhao, M. R. Ke, B. Y. Zheng, S. Huang, J. D. Huang, X. Li, Phthalocyanine aggregates as semiconductor-like photocatalysts for hypoxic-tumor photodynamic immunotherapy. *Nat. Commun.* **16**, 326 (2025).

27. J. Yu, J. Wu, J. Huang, C. Xu, M. Xu, C. Z. H. Koh, K. Pu, Y. Zhang, Hypoxia-tolerant polymeric photosensitizer prodrug for cancer photo-immunotherapy. *Nat. Commun.* **16**, 153 (2025).

28. K.-W. Lee, Y. Wan, Z. Huang, Q. Zhao, S. Li, C.-S. Lee, Organic optoelectronic materials: A rising star of bioimaging and phototherapy. *Adv. Mater.* **36**, 2306492 (2023).

29. N. Ma, J. Wang, H. Tang, S. Wu, X. Liu, K. Chen, Y. Zhang, X. Yu, The current advances in design strategy (indirect strategy and direct strategy) for type-I photosensitizers. *Adv. Sci.* **12**, e2413365 (2024).

30. F. Li, T. Li, K. Y. Li, M. Meng, X. Y. Guo, S. S. He, H. Y. Tian, Organic semiconducting sono-metallo-detonated immunobombs for ultrasensitized domestication of immunosuppressive cells. *Nano Lett.* **24**, 7340–7349 (2024).

31. S. He, J. Li, P. Cheng, Z. Zeng, C. Zhang, H. Duan, K. Pu, Enzymatically catalyzed molecular aggregation. *Angew. Chem. Int. Ed.* **60**, 19355–19363 (2021).

32. W.-J. Wang, R. Zhang, L. Zhang, L. Hao, X.-M. Cai, Q. Wu, Z. Qiu, R. Han, J. Feng, S. Wang, P. Alam, G. Zhang, Z. Zhao, B. Z. Tang, Charge-reversal polymer nano-modulators for photodynamic immunotherapy of cancer. *Nat. Commun.* **15**, 9999 (2024).

33. B. Ran, L. Ran, Z. Wang, J. Liao, D. Li, K. Chen, W. Cai, J. Hou, X. Peng, Photocatalytic antimicrobials: Principles, design strategies, and applications. *Chem. Rev.* **123**, 12371–12430 (2023).

34. J. Shen, T. W. Rees, L. Ji, H. Chao, Recent advances in Ruthenium(II) and Iridium(III) complexes containing nanosystems for cancer treatment and bioimaging. *Coord. Chem. Rev.* **443**, 214016 (2021).

35. Z. Li, B. Ding, J. Li, H. Chen, J. Zhang, J. Tan, X. Ma, D. Han, P. Ma, J. Lin, Multi-enzyme mimetic MoCu dual-atom nanozyme triggering oxidative stress cascade amplification for high-efficiency synergistic cancer therapy. *Angew. Chem. Int. Ed.* **64**, e202413661 (2024).

36. K. X. Teng, L. Y. Niu, Q. Z. Yang, Supramolecular photosensitizer enables oxygen-independent generation of hydroxyl radicals for photodynamic therapy. *J. Am. Chem. Soc.* **145**, 4081–4087 (2023).

37. H. Huang, S. Banerjee, K. Qiu, P. Zhang, O. Blacque, T. Malcomson, M. J. Paterson, G. J. Clarkson, M. Staniforth, V. G. Stavros, G. Gasser, H. Chao, P. J. Sadler, Targeted photoredox catalysis in cancer cells. *Nat. Chem.* **11**, 1041–1048 (2019).

38. C. Huang, C. Liang, T. Sadhukhan, S. Banerjee, Z. Fan, T. Li, Z. Zhu, P. Zhang, K. Raghavachari, H. Huang, In-vitro and in-vivo photocatalytic cancer therapy with biocompatible Iridium(III) photocatalysts. *Angew. Chem. Int. Ed.* **60**, 9474–9479 (2021).

39. Z. Fan, Y. Rong, T. Sadhukhan, S. Liang, W. Li, Z. Yuan, Z. Zhu, S. Guo, S. Ji, J. Wang, R. Kushwaha, S. Banerjee, K. Raghavachari, H. Huang, Single-cell quantification of a highly biocompatible dinuclear Iridium(III) complex for photocatalytic cancer therapy. *Angew. Chem. Int. Ed.* **61**, e202202098 (2022).

40. A. Dao, H. Wu, S. Wei, H. Huang, Novel Ru(II) complexes with multiple anticancer photoreactivity: Ligand exchange, photoredox catalysis, reactive oxygen generation and endoperoxide formation. *Phys. Chem. Chem. Phys.* **25**, 20001–20008 (2023).

41. A. Dao, S. Chen, L. Pan, Q. Ren, X. Wang, H. Wu, Q. Gong, Z. Chen, S. Ji, J. Ru, H. Zhu, C. Liang, P. Zhang, H. Xia, H. Huang, A 700 nm LED light activated Ru(II) complex destroys tumor cytoskeleton via photosensitization and photocatalysis. *Adv. Healthc. Mater.* **13**, e2400956 (2024).

42. H. Shi, O. W. L. Carter, F. Ponte, C. Imberti, M. A. Gomez-Gonzalez, F. Cacho-Nerin, P. D. Quinn, J. E. Parker, E. Sicilia, H. Huang, P. J. Sadler, A photodynamic and photochemotherapeutic platinum-iridium charge-transfer conjugate for anticancer therapy. *Angew. Chem. Int. Ed.* **63**, e202400476 (2024).

43. M. Li, K. H. Gebremedhin, D. Ma, Z. Pu, T. Xiong, Y. Xu, J. S. Kim, X. Peng, Conditionally activatable photoredox catalysis in living systems. *J. Am. Chem. Soc.* **144**, 163–173 (2021).

44. M. Li, Y. Xu, Z. Pu, T. Xiong, H. Huang, S. Long, S. Son, L. Yu, N. Singh, Y. Tong, J. L. Sessler, X. Peng, J. S. Kim, Photoredox catalysis may be a general mechanism in photodynamic therapy. *Proc. Natl. Acad. Sci. U.S.A.* **119**, e2210504119 (2022).

45. K. Zhou, L. Du, R. Ding, L. Xu, S. Shi, S. Wang, Z. Wang, G. Zhang, G. He, Z. Zhao, B. Z. Tang, Photocatalytic therapy via photoinduced redox imbalance in biological system. *Nat. Commun.* **15**, 10551 (2024).

46. C. Zhu, J. Cheng, H. Lin, Z. Yang, Y. Huang, F. Lv, H. Bai, S. Wang, Rational design of conjugated polymers for photocatalytic CO_2 reduction: Towards localized CO production and macrophage polarization. *J. Am. Chem. Soc.* **146**, 24832–24841 (2024).

47. S. Xia, H. Bai, E. Zhang, W. Yu, Z. Gao, F. Lv, Y. Huang, D. Zhu, S. Wang, Unexpected photocatalytic degeneration of NAD^+ for inducing apoptosis of hypoxia cancer cells. *CCS Chem.* **5**, 2324–2333 (2023).

48. K. X. Teng, L. Y. Niu, N. Xie, Q. Z. Yang, Supramolecular photodynamic agents for simultaneous oxidation of NADH and generation of superoxide radical. *Nat. Commun.* **13**, 6179 (2022).

49. T. Feng, Z. Tang, J. Shu, X. Wu, H. Jiang, Z. Chen, Y. Chen, L. Ji, H. Chao, A cyclometalated Ruthenium(II) complex induces oncosis for synergistic activation of innate and adaptive immunity. *Angew. Chem. Int. Ed.* **63**, e202405679 (2024).

50. F. Cao, H. Wang, N. Lu, P. Zhang, H. Huang, A photoisomerizable Zinc (II) complex inhibits microtubule polymerization for photoactive therapy. *Angew. Chem. Int. Ed.* **62**, e202301344 (2023).

51. J. Cheng, L. Li, D. Jin, Y. Zhang, W. Yu, J. Yu, J. Zou, Y. Dai, Y. Zhu, M. Liu, M. Zhang, Y. Sun, Y. Liu, X. Chen, A non-metal single atom nanozyme for cutting off the energy and reducing power of tumors. *Angew. Chem. Int. Ed.* **63**, e202319982 (2024).

52. R. Zhang, H. Xu, Y. Yao, G. Ran, W. Zhang, J. Zhang, J. L. Sessler, S. Gao, J.-L. Zhang, Nickel(II) Phototheranostics: A case study in photoactivated H_2O_2-enhanced immunotherapy. *J. Am. Chem. Soc.* **145**, 23257–23274 (2023).

53. C. Yang, J. Zhang, M. Chang, J. Tan, M. Yuan, Y. Bian, B. Liu, Z. Liu, M. Wang, B. Ding, P. Ma, J. Lin, NIR-activatable heterostructured nanoadjuvant CoP/NiCoP executing lactate metabolism interventions for boosted photocatalytic hydrogen therapy and photoimmunotherapy. *Adv. Mater.* **36**, 2308774 (2023).

54. L. Wu, H. Lin, X. Cao, Q. Tong, F. Yang, Y. Miao, D. Ye, Q. Fan, Bioorthogonal Cu single-atom nanozyme for synergistic nanocatalytic therapy, photothermal therapy, cuproptosis and immunotherapy. *Angew. Chem. Int. Ed.* **63**, e202405937 (2024).

55. A. S. Klymchenko, Fluorescent probes for lipid membranes: From the cell surface to organelles. *Acc. Chem. Res.* **56**, 1–12 (2022).

56. A. Prashar, C. Bussi, A. Fearns, M. I. Capurro, X. Gao, H. Sesaki, M. G. Gutierrez, N. L. Jones, Lysosomes drive the piecemeal removal of mitochondrial inner membrane. *Nature* **632**, 1110–1117 (2024).

57. C. Liu, J. Yan, M. Wu, W. Wang, L. Yuan, D. Tian, Y. Sun, R. Zhang, Self-immolative cationic iridium(III) complex-encapsulated nanoprodrug for enhanced chemo-photodynamic synergistic therapy of melanomas. *J. Am. Chem. Soc.* **147**, 27812–27821 (2025).

58. S. Wang, L. Gai, Y. Chen, X. Ji, H. Lu, Z. Guo, Mitochondria-targeted BODIPY dyes for small molecule recognition, bio-imaging and photodynamic therapy. *Chem. Soc. Rev.* **53**, 3976–4019 (2024).

59. I. Martínez-Reyes, N. S. Chandel, Mitochondrial TCA cycle metabolites control physiology and disease. *Nat. Commun.* **11**, 102 (2020).

60. D. G. Ryan, M. P. Murphy, C. Frezza, H. A. Prag, E. T. Chouchani, L. A. O'Neill, E. L. Mills, Coupling Krebs cycle metabolites to signalling in immunity and cancer. *Nat. Metab.* **1**, 16–33 (2018).

61. M. Bonora, M. R. Wieckowski, D. A. Sinclair, G. Kroemer, P. Pinton, L. Galluzzi, Targeting mitochondria for cardiovascular disorders: Therapeutic potential and obstacles. *Nat. Rev. Cardiol.* **16**, 33–55 (2018).

62. J. A. Amorim, G. Coppotelli, A. P. Rolo, C. M. Palmeira, J. M. Ross, D. A. Sinclair, Mitochondrial and metabolic dysfunction in ageing and age-related diseases. *Nat. Rev. Endocrinol.* **18**, 243–258 (2022).

63. W. Wang, S. Y. Yao, J. Luo, C. Ding, Q. Huang, Y. Yang, Z. Shi, J. Lin, Y. C. Pan, X. Zeng, D.-S. Guo, H. Chen, Engineered hypoxia-responsive albumin nanoparticles mediating mitophagy regulation for cancer therapy. *Nat. Commun.* **16**, 596 (2025).

64. B. Wang, H. Zhou, L. Chen, Y. Ding, X. Zhang, H. Chen, H. Liu, P. Li, Y. Chen, C. Yin, Q. Fan, A mitochondria-targeted photosensitizer for combined pyroptosis and apoptosis with NIR-II imaging/photoacoustic imaging-guided phototherapy. *Angew. Chem. Int. Ed.* **63**, e202408874 (2024).

65. Y. Peng, R. Mo, M. Yang, H. Xie, F. Ma, Z. Ding, S. Wu, J. W. Y. Lam, J. Du, J. Zhang, Z. Zhao, B. Z. Tang, Mitochondria-targeting AIEgens as pyroptosis inducers for boosting type-I photodynamic therapy of tongue squamous cell carcinoma. *ACS Nano* **18**, 26140–26152 (2024).

66. F. Tong, Y. Wang, Y. Xu, Y. Zhou, S. He, Y. Du, W. Yang, T. Lei, Y. Song, T. Gong, H. Gao, MMP-2-triggered, mitochondria-targeted PROTAC-PDT therapy of breast cancer and brain metastases inhibition. *Nat. Commun.* **15**, 10328 (2024).

67. L. Ren, J. Wan, X. Li, J. Yao, Y. Ma, F. Meng, S. Zheng, W. Han, H. Wang, Mitochondrial rewiring with small-molecule drug-free nanoassemblies unleashes anticancer immunity. *Nat. Commun.* **15**, 7664 (2024).

68. Y. Dong, S. Dong, C. Yu, J. Liu, S. Gai, Y. Xie, Z. Zhao, X. Qin, L. Feng, P. Yang, Y. Zhao, Mitochondria-targeting Cu_3VS_4 nanostructure with high copper ionic mobility for photothermoelectric therapy. *Sci. Adv.* **9**, eadi9980 (2023).

69. Z. Deng, H. Li, S. Chen, N. Wang, G. Liu, D. Liu, W. Ou, F. Xu, X. Wang, D. Lei, P.-C. Lo, Y. Y. Li, J. Lu, M. Yang, M.-L. He, G. Zhu, Near-infrared-activated anticancer platinum(IV) complexes directly photooxidize biomolecules in an oxygen-independent manner. *Nat. Chem.* **15**, 930–939 (2023).

70. M. J. Frisch, G. W. Trucks, H. B. Schlegel, G. E. Scuseria, M. A. Robb, J. R. Cheeseman, G. Scalmani, V. Barone, G. A. Petersson, H. Nakatsuji, X. Li, M. Caricato, A. V. Marenich, J. Bloino, B. G. Janesko, R. Gomperts, B. Mennucci, H. P. Hratchian, J. V. Ortiz, A. F. Izmaylov, J. L. Sonnenberg, Williams, F. Ding, F. Lipparini, F. Egidi, J. Goings, B. Peng, A. Petrone, T. Henderson, D. Ranasinghe, V. G. Zakrzewski, J. Gao, N. Rega, G. Zheng, W. Liang, M. Hada, M. Ehara, K. Toyota, R. Fukuda, J. Hasegawa, M. Ishida, T. Nakajima, Y. Honda, O. Kitao, H. Nakai, T. Vreven, K. Throssell, J. A. Montgomery Jr., J. E. Peralta, F. Ogliaro, M. J. Bearpark, J. J. Heyd, E. N. Brothers, K. N. Kudin, V. N. Staroverov, T. A. Keith, R. Kobayashi, J. Normand, K. Raghavachari, A. P. Rendell, J. C. Burant, S. S. Iyengar, J. Tomasi, M. Cossi, J. M. Millam, M. Klene, C. Adamo, R. Cammi, J. W. Ochterski, R. L. Martin, K. Morokuma, O. Farkas, J. B. Foresman, D. J. Fox, Gaussian 16 (Revision C.01) (Gaussian Inc., 2019).

71. T. Lu, F. Chen, Multiwfn: A multifunctional wavefunction analyzer. *J. Comput. Chem.* **33**, 580–592 (2012).

72. Y. Niu, W. Li, Q. Peng, H. Geng, Y. Yi, L. Wang, G. Nan, D. Wang, Z. Shuai, Molecular Materials Property Prediction Package (Momap) 1.0: A software package for predicting the luminescent properties and mobility of organic functional materials. *Mol. Phys.* **116**, 1078–1090 (2018).

73. C. Cantó, K. J. Menzies, J. Auwerx, NAD^+ metabolism and the control of energy homeostasis: A balancing act between mitochondria and the nucleus. *Cell Metab.* **22**, 31–53 (2015).

74. K.-X. Teng, D. Zhang, B.-K. Liu, Z.-F. Liu, L.-Y. Niu, Q.-Z. Yang, Photo-induced disproportionation-mediated photodynamic therapy: Simultaneous oxidation of tetrahydrobiopterin and generation of superoxide radicals. *Angew. Chem. Int. Ed.* **63**, e202318783 (2024).

75. X. Zhang, L. Li, Y. Ren, M. Li, Y. Tang, Organic NIR-II nanofluorophore withultrahigh quantum yield for vessels imaging and fluorescence image-guided surgery. *Adv. Funct. Mater.* **35**, 2413341 (2025).

76. M. Li, Z. Lu, J. Zhang, L. Chen, X. Tang, Q. Jiang, Q. Hu, L. Li, J. Liu, W. Huang, Near-infrared-II fluorophore with inverted dependence of fluorescence quantum yield on polarity as potent phototheranostics for fluorescence-image-guided phototherapy of tumors. *Adv. Mater.* **35**, 2209647 (2023).

77. C. You, Y. Zhu, J. Zhu, Z. Xu, Q. Liu, L. Wang, W. Zhang, J. Hou, D. Wang, B. Z. Tang, Strength in numbers: A giant NIR-II AIEgen with one-for-all phototheranostic features for exceptional orthotopic bladder cancer treatment. *Angew. Chem. Int. Ed.* **64**, e202417865 (2025).

78. S. Yang, J. Zhang, Z. Zhang, R. Zhang, X. Ou, W. Xu, M. Kang, X. Li, D. Yan, R. T. K. Kwok, J. Sun, J. W. Y. Lam, D. Wang, B. Tang, More is better: Dual-accept or engineering for constructing second near-infrared aggregation-induced emission luminogens to boost multimodal phototheranostics. *J. Am. Chem. Soc.* **145**, 22776–22787 (2023).

79. F. Tong, Q. Yang, , Z. Hu, S. Zhu, R. Ma, H. Ma, Z. Ma, H. Wan, T. Zhu, Z. Jiang, W. Liu, L. Jiao, H. Sun, Y. Liang, H. Dai, Donor engineering for NIR-II molecular fluorophores with enhanced fluorescent performance. *J. Am. Chem. Soc.* **140**, 1715–1724 (2018).

80. Y. Yuan, Z. Feng, S. Li, Z. Huang, Y. Wan, C. Cao, S. Lin, L. Wu, J. Zhou, L.-S. Liao, J. Qian, C.-S. Lee, Molecular programming of NIR-IIb-emissive semiconducting small molecules for in vivo high-contrast bioimaging beyond 1500 nm. *Adv. Mater.* **34**, e2201263 (2022).

81. Y. Liu, J. Liu, D. Chen, X. Wang, Z. Zhang, Y. Yang, L. Jiang, W. Qi, Z. Ye, S. He, Q. Liu, L. Xi, Y. Zou, C. Wu, Fluorination enhances NIR-II fluorescence of polymer dots forquantitative brain tumor imaging. *Angew. Chem. Int. Ed.* **59**, 21049–21057 (2020).

82. L. Hu, H. Xiao, L. Li, T. Hu, T. Guo, C. Wang, R. Liu, L. Ying, A fluorinated conjugated polymer allows for enhanced NIR-II fluorescence imaging-guided photothermal therapy. *Adv. Healthc. Mater.* **e02069**, (2025).

83. Z. Zhang, X. Fang, Z. Liu, H. Liu, D. Chen, S. He, J. Zheng, B. Yang, W. Qin, X. Zhang, C. Wu, Semiconducting polymer dots with dual-enhanced NIR-IIa fluores-cence for through-skull mouse-brain imaging. *Angew. Chem. Int. Ed.* **59**, 3691–3698 (2020).

84. P. Chen, F. Qu, S. Chen, J. Li, Q. Shen, P. Sun, Q. Fan, Bandgap modulation and lipid intercalation generates ultrabright D–A–D-based zwitterionic small-molecule nanoagent for precise NIR-II excitation phototheranostic applications. *Adv. Funct. Mater.* **32**, 2208463 (2022).

85. S. Song, Y. Zhao, M. Kang, F. Zhang, Q. Wu, N. Niu, H. Yang, H. Wen, S. Fu, X. Li, Z. Zhang, B. Tang, D. Wang, An NIR-II excitable AIE small molecule with multimodal phototheranostic features for orthotopic breast cancer treatment. *Adv. Mater.* **36**, 2309748 (2024).
